# Reporting of Safety Events during Anti-VEGF Treatment: Pharmacovigilance in a Noninterventional Trial

**DOI:** 10.1155/2020/8652370

**Published:** 2020-10-06

**Authors:** Focke Ziemssen, Thomas Hammer, Matthias Grueb, Bettina Mueller, Hüsnü Berk, Maria-Andreea Gamulescu, Jessica Voegeler, Joachim Wachtlin, OCEAN Study Group

**Affiliations:** ^1^Centre for Ophthalmology, Eberhard-Karls-University Tuebingen, 72076 Tuebingen, Germany; ^2^Augenzentrum Halle, Dessauer Str. 194, 06118 Halle, Germany; ^3^Universitätsklinik und Poliklinik für Augenheilkunde der Martin-Luther-Universität Halle-Wittenberg, Ernst-Grube-Str. 40, 06112 Halle, Germany; ^4^Augenarztpraxis Villa Im Lindengarten, Breisach, Germany; ^5^Novartis Pharma GmbH, 90429 Nuremberg, Germany; ^6^St. Elisabeth Klinik Hohenlind, Cologne, Germany; ^7^Universitätsaugenklinik Regensburg, Franz-Josef-Strauss Allee 11, 93051 Regensburg, Germany; ^8^Departement of Ophthalmology, Sankt-Gertrauden Krankenhaus, Paretzer Str. 12, 10713 Berlin, Germany; ^9^MHB Medizinische Hochschule Brandenburg, Neuruppin, Germany

## Abstract

**Aim:**

The prospective, noninterventional OCEAN study assessed the safety of intravitreal ranibizumab injections for treatment of neovascular age-related macular degeneration, diabetic macular edema, and retinal vein occlusion under real-world conditions in Germany.

**Methods:**

Adults receiving ≥1 ranibizumab (0.5 mg) injections were recruited by 369 ophthalmologists and followed for 24 months. Information on adverse events (AEs) was reported by the treating physician or detected by the data management team. Collected information included observed AE, AE start and end date, intensity, causal relationship, outcome, severity, suspected drug, and actions taken.

**Results:**

2,687 AEs were reported for 1,176 of the 5,781 patients who had received a total of 32,621 injections: 27.4% nonserious AEs, 30.3% serious AEs, 27.3% nonserious adverse drug reactions (ADRs), and 15.0% serious ADRs. Most patients reported no AEs (79.7%) or only 1 AE (10.3%). AEs were most commonly reported in the Medical Dictionary for Regulatory Activities (MedDRA) System Organ Class (SOC) Eye disorders (9.4% of patients) and General disorders and administration site conditions (5.8%). The most frequent AEs by MedDRA preferred term (PT) were visual acuity reduced (3.5% of patients), intraocular pressure increased (2.5%), and drug ineffective (2.1%). AEs occurred most frequently after 3 or 4 injections (1,129 of 2,687 AEs). The proportion of AEs in the SOC Eye disorders decreased slightly with increasing number of injections, from 39.8% of events after 1 or 2 injections to 29.1% after 5 or more injections. Rates of the most frequently reported PTs did not show any consistent increase with increasing number of injections. A decrease in overall AE rates was observed over the study course.

**Conclusions:**

The results did not raise any new safety concerns for ranibizumab. The findings allow conclusions to be drawn on how pharmacovigilance data can be collected even more effectively in real-world studies to facilitate discussion on benefit-risk ratio.

## 1. Introduction

A number of studies have addressed the safety of vascular endothelial growth factor (VEGF) inhibition for the treatment of neovascular age-related macular degeneration (nAMD), diabetic macular edema (DME) [1–3], and retinal vein occlusion (RVO) [4, 5]. Although anti-VEGF drugs for the treatment of retinal conditions are in general well tolerated, a number of ocular adverse events (AEs) are known to occur after intravitreal injections [6, 7]. Regarding safety in ophthalmology, vision is a precious asset, and negative impacts of ocular treatments on general health or other areas of the body have to be considered as well, especially for older patients. It is important to be aware that certain comorbidities have been associated with the risk of the treatment indications [8, 9]; for example, cardiovascular risk factors cause venous occlusion [10], or poorly controlled blood pressure/blood sugar contributes to DME [11]. This makes older patients with neovascular or edematous retinal disease—especially the sicker ones outside the selection of randomized controlled trials (RCTs)—“risk patients” in routine clinical practice [12].

Patient safety must always be considered during drug design, development, and use. For drugs with intravitreal application, the early stages of development should aim to minimize systemic exposure and to reduce the risks of ocular side effects, particularly intraocular infection [13]. Nonocular AEs have also been reported for anti-VEGF drugs, including elevated blood pressure, myocardial infarction, and stroke [14, 15].

Despite the huge number of treatments carried out worldwide every year [16, 17], the small ophthalmology community is faced with the following methodological problems. First, prospective RCTs are designed to detect the efficacy of a drug and are not sufficiently powered to detect rare AEs, and even meta-analyses of RCTs cannot overcome this issue [18–20]. Second, studies in internal medicine use different analysis sets to examine specific subgroups in order to achieve the necessary sensitivity and to detect particularly at-risk subgroups [1]. Documentation of AEs and adverse drug reactions (ADRs) in observational studies is crucial for the safety profile of a study drug. According to the International Council for Harmonisation of Technical Requirements for Pharmaceuticals for Human Use (ICH) and Harmonised Tripartite Guideline on Clinical Safety Data Management (E2A), an AE is defined as “any untoward medical occurrence in a patient or clinical investigation subject administered a pharmaceutical product and which does not necessarily have to have a causal relationship with this treatment.” An ADR, on the other hand, is defined as “a response to a drug which is noxious and unintended and which occurs at doses normally used in man for prophylaxis, diagnosis, or therapy of disease or for modification of physiological function” (ICH 1994), i.e., a causal relationship is at least suspected by the medical practitioner. Thus, ADRs are a subset of all AEs.

Second, data from health care research can provide indications of a safety signal. However, there are important limitations. The selection of a drug or therapy decision is decisively influenced by sociocultural factors such as the assumption of costs [21–24]. Although the analysis of accounting data can make an important contribution here [25], many questions about the possible causal relationships of these risks cannot be answered. Furthermore, the increased risk of AEs and ADRs in the aged population is characterized by age-related changes in drug pharmacodynamics and pharmacokinetics and a large number of different risk factors and comorbidities [26].

Third, when a drug is administered intravitreally, it is important, but not always easy, to make a clear distinction between complications of the method of application and the side effects of the active ingredients. Intolerance of the disinfectant can be attributed to the intravitreal procedure. For intraocular inflammations, on the other hand, it is much more difficult to distinguish between immunogenic inflammatory reactions related to the active substance or silicone oil of the syringe and a carryover of bacteria from the conjunctiva into the eye due to the intravitreal procedure [27].

Finally, interpretation of safety outcomes becomes even more difficult when the absolute incidences of discussed effects are rarer than the reported spontaneous incidence [28]. Changing personnel and inertia in reporting makes comprehensive assessment of AEs difficult [29, 30]. Generally, safety reporting is requested but remains at the physician's discretion, and underreporting of AEs is a major problem [29–31]. Events of little relevance to the therapeutic area, rare events, and events with no suspected causal relationship are often not reported, making safety reporting incomplete [32, 33].

The present analysis examines the safety in a large noninterventional study, aiming to provide further estimates for the safety profile of VEGF inhibition in ophthalmological indications. The analysis was also undertaken to question and improve the methods for the acquisition of safety signals within the framework of Phase IV studies.

## 2. Materials and Methods

### 2.1. Study Design

The prospective, multicenter, noninterventional OCEAN study (“Observation of treatment patterns with LuCEntis and real life ophthalmic monitoring, including optional OCT in Approved iNdications”; NCT02194803, ClinicalTrials.gov) was designed to assess the outcomes of intravitreal ranibizumab injections in adults with nAMD, DME, RVO, or myopic choroidal neovascularization (mCNV) in routine clinical practice in Germany. The study was conducted between December 2011 and December 2016, with an observational period of up to 24 months per patient. The study documents including the observational plan were implemented in accordance with the Declaration of Helsinki. Ethics committee approval was obtained, and each patient provided written informed consent. All injections and examinations were performed at the treating physician's discretion. Data including details of ranibizumab injections, visual acuity outcomes, and AEs were provided by the study physicians. Details of the study design, in particular, possible predisposing baseline demographics have been published previously [34].

Safety-related data were collected in a dedicated safety database. This database was reconciled with the Novartis pharmacovigilance database (after clarification of inconsistent data entries with study sites) and completed in August 2017. The collected data did not include AEs that occurred after the observation period, though delayed ADRs and end-of-use ADRs were evaluated.

At study start, only paper case report forms (CRFs) were available. Documentation by electronic CRF was added as an alternative to paper documentation about one year after the study start. Most physicians documented data on paper CRFs. The AE reporting form of the CRF included the following information: observed AE, start and end date of AE, intensity, causal relationship with ranibizumab, outcome, and severity, suspected drug, and actions taken. Additional information for serious AEs (SAEs) included concomitant medication, concomitant diseases, and cause of death in case the patient died. All AEs, both ocular and nonocular events, were documented in the CRF.

### 2.2. Classification of Adverse Events

For the analysis of the safety data of the OCEAN study, classifications of AEs based on the ICH E2A [35] were defined as follows: an AE was defined as any unfavorable and unintentional sign or symptom or any disease occurring in a chronological relationship with the use of ranibizumab in this study, irrespective of whether a causal relationship with this medicinal product was assumed. In addition, all of the following events and/or situations were considered as AEs, irrespective of whether a clinical symptom appeared or not: interaction with other substances or products, exposure during pregnancy or while breast-feeding, use by the father before or at the time of fathering a child, inadequate or lack of efficacy, inadvertent or deliberate overdose, abuse and misuse, dependence, withdrawal/discontinuation/rebound phenomena, medication and administration errors, progression or aggravation of the primary disease, positive effects (unexpected), occupational exposure, and quality defects.

AEs and ADRs were differentiated into serious AEs (SAE) or ADRs (sADR) and nonserious AEs (nsAE) or ADRs (nsADR). SAEs or sADRs were defined as events that were fatal or life-threatening, necessitated inpatient hospitalization or prolongation of existing hospitalization, resulted in inability to work, persistent or significant disability, or invalidity, resulted in a congenital anomaly or a birth defect, or were medically significant. In turn, nsAEs and nsADRs were defined as AEs that did not fulfil these criteria for SAEs.

An (S) AE was classified as an (s) ADR if the causality to ranibizumab therapy was assessed by the physician as “definite,” “probable,” or “possible,” or if the causality was “not assessable,” or if assessment of causality was missing. A medical assessment of causality had to be recorded by the physician for each reported AE.

### 2.3. Data Analysis

AEs were coded using version 19.1 of the Medical Dictionary for Regulatory Activities (MedDRA), and drugs were coded using the WHO drug dictionary (DD) version 12/2016.

AEs were presented by general AE incidence tables related to all patients valid for the safety population. These tables included patient-based and event-based analyses of incidences of AEs, analyses by category of AE, number of AEs per patient (absolute and relative frequencies), and classification of AEs by duration, intensity, outcome, causality, and action taken. In addition, listings of nsAEs, SAEs, deaths, and pregnancies were generated.

The safety evaluation set (SES) included patients with documentation of at least one injection of ranibizumab 0.5 mg during the study and for whom follow-up information regarding safety was available (≥1 follow-up visit or AE occurrence or premature discontinuation).

Additionally, a time-to-event Cox proportional hazard regression was performed to assess the effect of gender, age, baseline best-corrected visual acuity, BMI, indication (nAMD, DME, or RVO), and physician location (hospital-based or private practice) on the occurrence of any AE. This analysis was limited to patients with no missing data in the respective variables.

## 3. Results

### 3.1. Patient Population and Study Period

A total of 369 ophthalmologists (study sites) participated in the OCEAN study. All 5,781 patients treated in the study were included in the SES for the current analysis. The SES included 3,726 patients with nAMD, 1,250 patients with DME, 764 patients with RVO, and 40 patients with mCNV. A list of the participating OCEAN study sites is provided in Supplementary Table S1.

The mean age of patients included in the analysis was 74.6 years. Slightly more female patients (56.0%) than male patients (43.8%) were included. The patients received between 1 and 24 injections over the observation period, with a mean of 5.7 injections (Table 1).

### 3.2. Occurrence of Adverse Events

During the study, a total of 2,687 AEs (nonserious or serious) were reported for 1,176 of the 5,781 patients in the safety population (20.3%). Of these AEs, 27.4% was nsAEs, 30.3% was SAEs, 27.3% was nsADRs, and 15.0% was sADRs. From a patient-based perspective, 7.8% of patients had nsAEs, 7.2% had SAEs, 8.6% had nsADRs, and 3.7% had sADRs. Overall, most patients reported no AEs (79.7%) or only 1 AE (10.3%). A total of 32,621 injections were administered. The results did not raise any new safety signals for ranibizumab.

Little difference in AE documentation was observed between male patients (21.2% with AEs) and female patients (19.8% with AEs). Similarly, documentation of AEs was comparable among patients of different age groups, with AEs reported for 19.0% of patients ≤70 years of age, 20.6% of patients >70–<80 years of age, and 21.2% of patients ≥80 years of age. When assessing AE rates by age and gender combined, also, no major differences were seen across the groups.

AEs were most commonly reported in the MedDRA System Organ Class (SOC) Eye disorders (9.4% of patients), followed by general disorders and administration site conditions (5.8%). Events in the SOC Surgical and medical procedures occurred in 2.6% of patients only. The most frequent AEs by MedDRA preferred term (PT) were visual acuity reduced (3.5% of patients), intraocular pressure increased (2.5%), and drug ineffective (2.1%).

The most commonly reported AEs by category are presented in Table 2. A complete list of all reported AEs can be found in Supplementary Table S1.

Almost half of all AEs were classified as mild (21.9% of all AEs) or moderate (23.1%), while 17.0% were classified as severe (remaining AEs: no intensity provided or intensity unknown). Most SAEs were classified as severe (33.4%) or moderate (24.3%).

Regarding the outcome of the AEs, most of the AEs with a known outcome were classified as recovered (28.6%), whereas 13.1% were ongoing, 13.0% improved, 6.1% fatal, 2.4% worsened, and 1.5% recovered with sequelae. The most frequently documented outcome for SAEs with a known outcome was recovered (27.7%), followed by improved (17.5%), fatal (15.3%), and ongoing (14.8%).

For the majority of AEs (71.9%), the duration was unknown (mostly not calculable due to missing start and/or end dates). The duration was 1–7 days for 8.9%, 8–14 days for 4.4%, 15–21 days for 2.3%, and >21 days for 12.5% of the AEs.

The full data set of all AEs reported in the OCEAN study (Supplementary Table S1), which was coded according to the MedDRA system by the study data managers, included a total of 613 different MedDRA PTs in 27 SOCs.

Certain age-dependent events were seen in several patients: 113 patients (2.0% of all patients) died, 51 patients (0.9%) experienced neoplasms (benign, malignant, and unspecified, including cysts and polyps), 43 patients (0.7%) experienced fall, 16 patients (0.3%) experienced myocardial infarction, and 2 patients (0.03%) experienced acute myocardial infarction. The patients' elderly mean age of 74.6 years in this study must be considered.

When examining the occurrence of events over time, the SOC Neoplasms (benign, malignant, and unspecified, including cysts and polyps) were least common between baseline and month 3 and after month 21 (6.7% and 8.3% of events, respectively) and were consistently slightly higher over the rest of the study period (range: 10.0%–20.0% of events). The PT fall was more common at study start (from baseline to month 3 and from month 3 to month 6, 18.2% of events each) and from month 16 to month 18 (20.5% of events), while during the remaining study period, the number of events was consistently lower (range: 4.5%–11.4%). No general patterns in the occurrence of myocardial infarction, acute myocardial infarction, or death were observed.

Though the occurrence of AEs in the SOC Gastrointestinal disorders were low (1.2% of all patients), they were examined in further detail due to the imbalance shown previously in the CATT study [36]. The related PTs included 2 patients (0.2%) with ascites and 1 patient (0.1%) each for the PTs diarrhoea, gastric ulcer, gastritis, gastrointestinal haemorrhage, intestinal perforation, large intestinal stenosis, loose tooth, nausea, and vomiting.

### 3.3. Adverse Event Incidences by Indication

**Figure 1:**
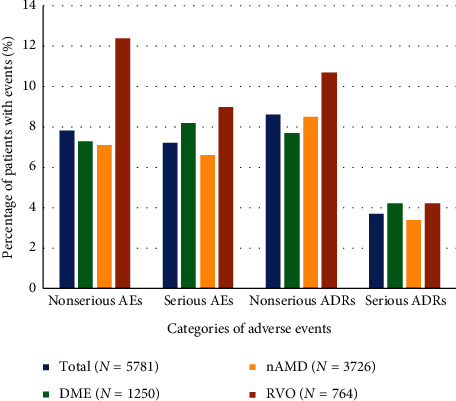
Incidence rates for adverse events by category across indications (nAMD, DME, and RVO). Nonserious AEs and ADRs occurred more frequently among RVO patients compared to nAMD and DME patients. Serious AEs and ADRs occurred slightly less frequently in nAMD patients than in RVO and DME. AE, adverse event; ADR, adverse drug reaction; DME, diabetic macular edema; nAMD, neovascular age-related macular degeneration; RVO, retinal vein occlusion.

The number of AEs, SAEs, and nsADRs slightly differed between the three main indications treated in the study (DME, nAMD, and RVO): the percentage of patients with nsAEs and with SAEs was slightly higher in RVO (12.4% and 9.0%, respectively) than in DME (7.3% and 8.2%, respectively) and nAMD (7.1% and 6.6%, respectively). Similarly, the percentage of nsADRs was higher in RVO (10.7%) than in nAMD (8.5%) and DME (7.7%). The percentage of patients with sADRs was similar for RVO (4.2%), DME (4.2%), and nAMD patients (3.4%) (Figure 1).

### 3.4. Adverse Event Occurrence by Number of Injections

AEs were additionally analyzed depending on the number of ranibizumab injections administered to the respective patient prior to the start of the AE. Overall, AEs occurred most frequently after 3 or 4 injections (1,129 of 2,687 AEs for 5,114 patients), followed by 5 or more injections (859 AEs for 2,912 patients) and 1 or 2 injections (596 AEs for 5,781 patients), while the number of injections the patient received was unknown for the remaining 103 AEs.

**Figure 2:**
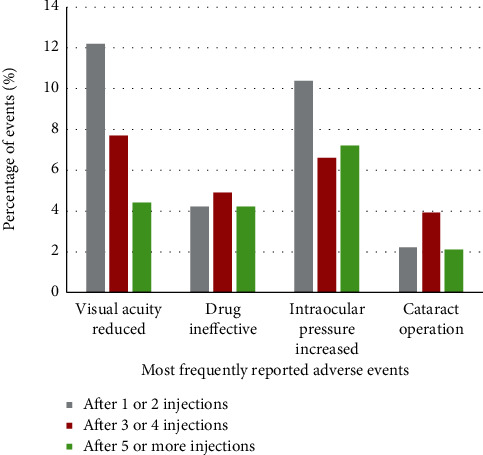
Incidence rates of most frequently reported adverse events stratified by how many injections of ranibizumab the patient received before adverse event (AE) occurred. None of the most frequently reported adverse events occurred more frequently with higher number of injections. Percentages are based on the total number of AEs (2687 AEs = 100%). Figure shows all events occurring with a rate of ≥2% overall.

When examining specific patterns in reported AEs by number of injections, the proportion of AEs in the MedDRA SOC Eye disorders decreased slightly with increasing number of injections, from 39.8% of events after 1 or 2 injections to 35.5% after 3 or 4 injections and 29.1% after 5 or more injections. Similarly, the rates of the most frequently reported MedDRA PTs did not show any consistent increase with increasing number of injections (Figure 2).

### 3.5. Reporting of Adverse Events

Hospital-based physicians reported AEs for 26.9% of their OCEAN patients, while physicians in private practice reported AEs for 19.2% of their patients. However, it must be noted that the vast majority of patients (4,938) was treated in private practices and only 843 in hospitals. Thus, not surprisingly, most patients with AEs (80.7%) were reported by private practices.

The delay between the start of an AE and its documentation was 30–<100 days for 23.9% of all AEs and 100–<300 days for 19.4%, followed by 300 and more days for 14.0%, 10–<30 days for 11.1%, 3–<10 days for 4.4%, and 0–<3 days for 3.3% of AEs. The delay was not calculable for the remaining 24% of AEs, mainly due to missing AE start dates. Many more AEs were reported on paper (77.0%) than electronically (23.0%). The delay between the start of an AE and its documentation was notably higher for paper-based reports than for electronic reports: more than 80% of reports with delays of >30 days originated from paper-based reporting. In contrast, 77.3% of all reports submitted within 2 days of an AE's start were received electronically.

While 86.9% of all AEs was documented directly by the physicians as AEs, 13.1% of AEs was detected as “hidden events” by the study data management team, i.e., were identified through physicians' free text entries or other hints in the study documentation and only subsequently after consultation with the study site, reported as AEs by the physicians. For most of these “hidden events,” the delay in documentation could not be calculated, mainly due to missing start dates for such AEs.

### 3.6. Adverse Event Rates over Time

**Figure 3:**
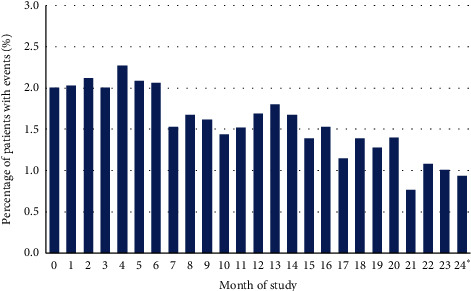
Overall incidence rates of adverse events starting at each month of the study. Calculation of percentages was based on number of patients not discontinued in the respective month. Patients with multiple adverse events within one month were counted only once. ^*∗*^An additional 77 events occurred after the 24-month study period. As the incidence rates are calculated based on the number of nondiscontinued patients, it was not possible to calculate the incidence rate for the AEs occurring after study end.

A decrease in overall AE rates was observed over the course of the study (Figure 3).

**Figure 4:**
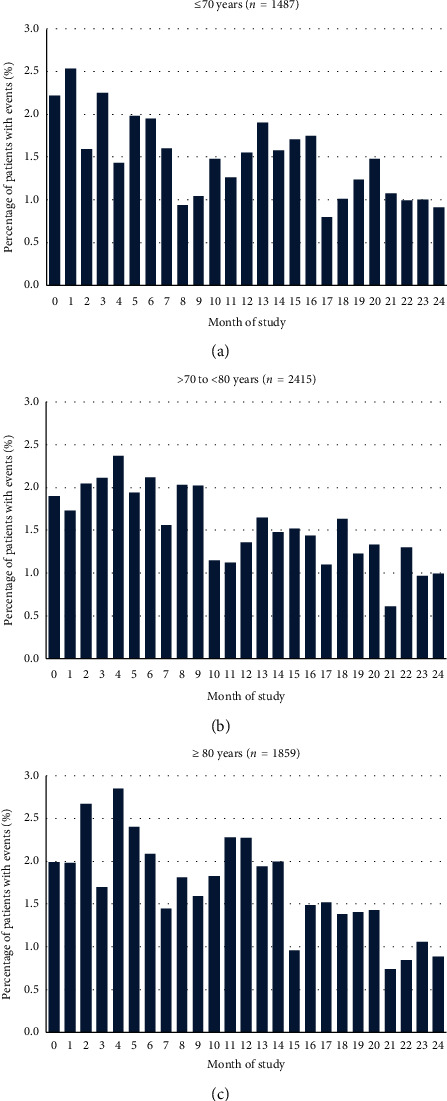
Incidence rates of adverse event for each month of the study, by age group. No consistent impact of the patients' age on the overall incidence rate of AEs was observed. Calculation of percentages was based on number of patients not discontinued in the respective month. Patients with multiple adverse events within one month were counted only once.

Furthermore, no consistent age-dependent trends were observed (Figure 4).

The overall number of events and the occurrence rates of the most frequently reported AEs over the course of the observational period is shown in Table 3. The 3 monthly incidence rates for these AEs did not increase notably or consistently over time.

### 3.7. Ocular Adverse Events

**Figure 5:**
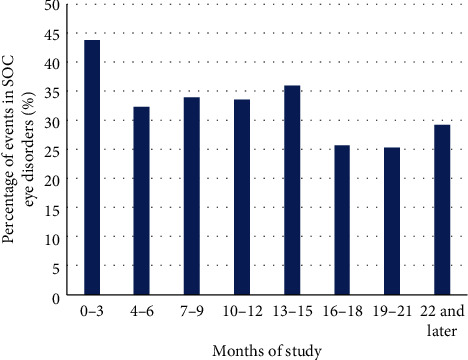
Percentages of adverse events in the MedDRA SOC Eye disorders, over the course of the study. No increase in the proportion of eye disorders was observed over the course of the study. Percentages were calculated based on the total number of events in the respective time period. AE, adverse event; MedDRA, Medical Dictionary for Regulatory Activities; SOC, System Organ Class.

The incidence rate of AEs in the MedDRA SOC Eye disorders was higher in the first three months of the study but remained quite constant in the remaining observation period (Figure 5).

### 3.8. Risk Factors and Comorbidities

Preexisting anamnestic risk factors and comorbidities were documented at the start of the OCEAN study. The study CRF prespecified a list including the following risk factors for the physicians to select diabetes mellitus (36.10% of 5,781 patients), neovascular disease of the other eye (23.87%), hypercholesterolaemia (12.18%), myocardial infarction (5.64%), hyperlipidaemia (5.24%), coronary artery disease in family (4.90%), and apoplexy (4.31%).

The prevalence rates of these risk factors were compared between patients who experienced AEs over the course of the OCEAN study (*n* = 1,176) and patients without any AEs (*n* = 4,605). No major differences between the two groups were seen, and the differences in the prevalence of risk factors did not explain the observed AEs.

When assessing the documented risk factors by the patients' age group (≤70 years (*n* = 1,487), >70–<80 years (*n* = 2,415), and ≥80 years (*n* = 1,859)), no major age-dependent differences were seen for myocardial infarction, apoplexy, coronary artery disease in the family, hypercholesterolaemia, and hyperlipidaemia. The prevalence of neovascular disease of the other eye increased with age, from 15% among the ≤70 year olds to 31% among the ≥80 year olds. On the other hand, the prevalence of diabetes mellitus was highest in the ≤70 years age group (53%) and lowest in the ≥80 years group (24%). However, it must be noted that, due to the OCEAN patients' relatively high mean age, all three analyzed age subgroups are of comparably older age.

The risk factors were also analyzed by geographical region, by analyzing the postal codes of the reporting study sites. The differences between the regions were only small, though rural areas showed slightly higher risk factor prevalences compared to city, urban, and suburban areas.

### 3.9. Regression Analysis

A Cox proportional hazard regression was performed using the complete data from 5,004 patients including 1,050 events for first occurrence of any type of AE. The regression included sex, baseline visual acuity, age, BMI, indication (nAMD, DME, or RVO), and physician location (hospital-based or private practice) (Table 4).

The regression generally showed the same patterns that were observed in the descriptive analysis. One notable difference, however, was the influence of age that was not evident when examining descriptive statistics alone. A possible explanation for this being is the association between age and the indication subgroups where the oldest subgroup (nAMD) reported the lowest proportion of AEs (7.1% nsAEs, 6.6% SAEs).

### 3.10. Comparison of Reported Rates with Clinical Trials

The reported incidence rates of selected ocular AEs in the OCEAN study were compared to those reported in clinical trials in the respective indications.

The incidence rates of selected AEs among the AMD patients of the OCEAN study were compared to those of the randomized, controlled trial TREND, which assessed efficacy and safety of 0.5 mg ranibizumab used according to a treat-and-extend (T&E) regimen compared to monthly injections [37]. Visual acuity reduced, and retinal haemorrhage occurred slightly less frequently in the OCEAN study than in the TREND study (Supplementary Figure S1). This difference was even more pronounced for intraocular pressure increased, cataract, conjunctival haemorrhage, and nasopharyngitis, which were reported much less frequently in OCEAN than in TREND. When comparing results, the difference in study design needs to be considered, as the TREND study employed a T&E regimen over 12 months with a mean of 8.7 injections, while the OCEAN study used a *prorenata* (PRN; treatment as needed) regimen over 24 months with a mean of 4.5 injections in the first 12 months and 5.7 injections over the full 24 months.

The incidence rates of these ocular AEs were also compared between the DME patients of the OCEAN study and those in the PRN group of the 24-month, randomized, controlled, trial RETAIN, which compared T&E administration of ranibizumab 0.5 mg (with and without laser) to a PRN regimen [38] (Supplementary Figure S2). Intraocular pressure increased, cataract, conjunctival haemorrhage, and nasopharyngitis were reported notably less frequently in OCEAN than in the RETAIN study. Retinal haemorrhage was reported for 2 DME patients in OCEAN (0.2%), while it did not occur in the PRN group of the RETAIN study. The PT visual acuity reduced occurred in 2.4% of patients in the OCEAN study based on MedDRA coding, and visual acuity reduced of ≥10 letters occurred in 3.4% of patients in the PRN group of the RETAIN study, though it must be noted that comparability is limited as visual acuity reduced was measured differently, and the study physicians did not always adhere to a uniform definition of terms.

In addition, the incidences of these AEs were compared between the RVO patients of the OCEAN study and the 24-month, open-label, single-arm CRYSTAL study, which assessed efficacy and safety of an individualized regimen of ranibizumab 0.5 mg driven by stabilization criteria [39] (Supplementary Figure S3). Visual acuity reduced occurred at similar rates in the two studies. Intraocular pressure increased, cataract, conjunctival haemorrhage, and nasopharyngitis were less frequently reported in OCEAN than in the CRYSTAL study. For retinal haemorrhage, no incidence rate was published for CRYSTAL; therefore, no comparison can be made.

## 4. Discussion

### 4.1. Status of Safety Reporting in the Noninterventional OCEAN Study

The current evaluation of the largest German noninterventional eye study can make a significant contribution to specifying the safety profile of ranibizumab. Over the course of the 2-year OCEAN study, 20.3% of patients experienced AEs, including 7.2% of patients with SAEs and 3.7% of patients with sADRs. During the entire study, 2,687 AEs with 613 different MedDRA PTs in 27 SOCs were reported. AEs were most commonly reported in the MedDRA SOC Eye disorders (9.4% of patients). When assessing the reported AEs separately for the main study indications, RVO patients tended to show slightly higher incidence rates compared to DME and nAMD patients. No major differences between male and female patients or between age groups in the reporting of AEs were found. The overall AE incidence rate, the number of AEs per 3 monthly intervals, the incidence rates of the most frequently reported AEs, and the incidence rate of events in the SOC Eye disorders tended to decrease over the course of the study. In line with this, no increase in the overall AE incidence rate was seen with increasing number of ranibizumab injections. Furthermore, the proportion of AEs in the MedDRA SOC Eye disorders decreased slightly with increasing number of injections.

Our analysis identified several issues with the reporting of AEs and comorbidities in noninterventional studies. Not only the frequency of the reported events but also the nature of the observations provides some insight into how the perspective and vigilance of the practitioners could have influenced the reporting behavior. Generally, AEs tend to be underreported in routine medical care when compared to controlled clinical trials in which AEs are rigorously documented, with a systematic review finding a median underreporting rate of 94% (interquartile range: 82–98%) [40]. As the OCEAN study was noninterventional, safety reporting was performed at the physicians' discretion, and no monitoring was performed. Therefore, the level of completeness of the safety data cannot be determined. In the absence of monitoring, the reported safety data were evaluated indirectly, by plausibility checks included in the study database, and participating physicians were alerted to obvious errors or implausible data entry, in order to correct these. Nevertheless, the documented incidences of AEs and SAEs over two years were lower than those published from clinical trials in the corresponding indications, likely due to underreporting in the noninterventional design. Similar underreporting of delayed ADRs and end-of-use ADRs can also be assumed. AEs were most commonly reported in the MedDRA SOC Eye disorders (9.4% of patients), and nonocular AEs were underreported, suggesting that participating ophthalmologists were less likely to consider or recognize AEs outside their specialty and mainly reported ophthalmological AEs, although the CRF asked physicians to document all AEs. Furthermore, the causal relationship of an AE to ranibizumab (relatedness) was determined by the treating physicians, possibly introducing some bias to the data. Future studies could consider the use of algorithms for determining causality [41]. The organizational form of the practice was also shown to have an influence on AE reporting, as hospital-based physicians reported AEs for a higher proportion of their patients compared to physicians in private practice. The reason behind this documentation difference might be that physicians at larger institutions have more experience with clinical studies and are therefore more familiar with AE reporting.

To assess the reliability of documented risk factors and comorbidities, the rates of selected comorbidities recorded during OCEAN were compared to published prevalence rates:

- The reported rates for diabetes mellitus were higher in this study (36.1%, OCEAN population's mean age of around 75 years) than those published for a comparable age group in Germany (∼20%, for the 70–79 years age group) [42–44]. Obviously, this is due to the fact that the study included a large proportion of DME patients. Among the nAMD and RVO patients in OCEAN, diabetes was recorded as a comorbidity for around 19% of patients.
- The prevalence of myocardial infarction in OCEAN was 5.6%, which is lower than a published prevalence rate for myocardial infarction of around 10% in the 70–79 years age group in Germany [45].
- Apoplexy (stroke) was reported for 4% of OCEAN patients, which is a lower prevalence than the 7% estimated for 70–79 year olds in the general population in Germany [46].

The reported rates of two of these three selected risk factors in OCEAN are below the prevalence rates estimated for the general population of comparable age in Germany. This finding supports the notion that ophthalmologists reporting AEs and comorbidities in ophthalmologic noninterventional studies do not always document all relevant events and risk factors, particularly those not directly related to ophthalmology. Two explanations for this observation can be considered. One is that health care data suggest that there is a gap in health care with increasing age and levels of care, and that some older, presumably sick patients are unable to find their way to ophthalmic therapy [47]. The other is that the preexisting anamnestic data of patients are incomplete, and even the electronic documentation of physicians has gaps about relevant previous illnesses [48]. No major difference in the prevalence of certain risk factors and comorbidities was seen between patients who experienced AEs during the study and patients without AEs.

### 4.2. Strengths and Limitations

A large number of sites and patients were included in the OCEAN study in order to obtain a realistic insight into the real-life treatment situation in Germany. The large sample size in combination with its prospective design render it less sensitive to selection bias in comparison to smaller and retrospective studies. However, observational artefacts and selection over time, i.e., loss to the follow-up, might contribute to a relevant amount of bias [49]. Obvious limitations in the reported safety data were seen for parameters such as AE outcome and AE duration, which were reported as unknown for a large proportion of events. The majority of AEs were reported with a delay of at least 30 days, though for many AEs, the delay could not be determined due to an unknown start date. A likely reason for the notable delay was the fact that 77% of AEs in the OCEAN study were reported on paper rather than electronically. Furthermore, it is important to note that 13% of AEs in the study was detected by the data management team as “hidden events”, i.e., events that were not documented by the physicians as AEs but were mentioned in other contexts within the CRF. For such events, the reporting delay was even higher, as they were only reported after the physician was contacted.

The heterogeneous patient population likely reflects the general population's heterogeneous risk profile more closely than clinical trials. The documentation of AEs was not as stringent as in clinical trials; hence, a number of AEs might have been missed.

In other trials, a number of associations were observed secondary to selection. For instance, treatment was related to social level or education, and intensity of treatment was related to comorbidities and general health status [50–52]. The completeness and quality of the long-term care differed among racial groups and people with different gross incomes [53].

Furthermore, it should be noted that ranibizumab is a product with a comparably short half-life and, therefore, fewer potential effects on systemic VEGF levels [54, 55]. Therefore, safety reporting issues in noninterventional studies may be even more problematic when aiming to establish a product's long-term safety profile under real-world conditions.

A more general limitation of recording safety information in this manner is that causal connection between treatment or different drugs and AE occurrence cannot be made. Neither ADRs nor AEs can provide adequate evidence for the causal inference. Due to the nature of spontaneous reporting, such events can only provide hypothesis-generating signals for potential causal relationships that may require more rigorous investigation. However, noninterventional studies such as OCEAN provide real-life insights, for example, how ophthalmologists evaluate and handle AEs in routine clinical practice.

This study did not collect data to assess the prevention of AEs, which would be of clinical relevance, especially given the elderly study population. However, clinical experience demonstrates that, when weighing the risks and benefits, many patients, including elderly patients, prioritize preserving their vision and quality-of-life over the avoidance of potential AEs. Future research could look into AE preventability, though ultimately weighing the risks and benefits comes down to shared decision-making between the physician and patient.

### 4.3. Recommendations

Although physicians in Germany are obligated to report AEs, underreporting is likely in noninterventional studies, as safety reporting may not be compulsory but is performed at the physician's discretion. On the other hand, clinical trials also have limitations, including patient selection, smaller sample size, and limited follow-up. Thus, caution is mandatory when analyzing the safety profile of intraocular drugs. Especially in ophthalmic diseases, the advanced age of many patients can often lead to a large variety of comorbidities. Ophthalmologists may not always be aware of all such comorbidities and risk factors, and patients may not remember to mention all of them. Furthermore, the limited life expectancy of elderly patients must be taken into account.

Concrete suggestions for safety reporting in noninterventional studies, based on our study results are

- Adoption of risk scores for pretreatment assessment
- It is helpful to assess a patient's risk profile in more detail prior to treatment and/or study enrolment. Big data analyses could provide the possibility to merge a large amount of clinically available information and accompanying parameters into key indications. Patients with ophthalmic diseases are often of advanced age and have a large variety of comorbidities, which can be overlooked by ophthalmologists. Future research should therefore aim to include health insurance data for a comparison of study results to patients without exposure to the study drug or treatment, allowing adjustment for age and disease-related comorbidities.
- Involvement of patients
- Direct communication channels to the study patients (or a subsample) would be advantageous to obtain further and more direct information, particularly regarding issues such as loss of the follow-up, adherence, and underreporting of AEs.
- Use of electronic notifications and digital channels
- The current analysis showed that electronic data collection is faster and more complete compared to paper-based data collection. The additional time and effort associated with paper-based documentation likely discourages many ophthalmologists from reporting observed events consistently and promptly. Future phase IV studies should take this into account and rely on electronic data capture only/mainly.
- Incentivization and transparency
- The knowledge of safety and side effects has a great value for patients and doctors. Currently, there are too few incentives to compile a scientifically sound picture of potential rare side effects. Because noninterventional studies are still undervalued, there is a lack of comprehensive data collections that can be adjusted for baseline criteria. It should not be permissible for health insurance companies to refuse to evaluate anonymized big data records or to prevent the participation in studies required by law within selective contracts. Indirect measures from the epidemiological toolbox can be used to check for the extent of underreporting and whether the data are representative. Health care research data without patient exposure to study drug or treatment might be a good comparator, allowing adjustment to age-/disease-related morbidities.
- Efforts to improve data quality
- Implementation of monitoring and source data verification should also be considered for noninterventional trials to allow an estimation of data quality and completeness. Despite the need for a large number of cases, efforts to ensure good reporting quality and knowledge of nonocular diseases must not be neglected. Indirect measures can be used to check for the extent of underreporting.

## 5. Conclusions

Physicians must aim for an even balance when discussing a treatment's or a study's benefits and disadvantages. Neither exaggeration nor trivialization of risks is helpful for a patient when discussing the initiation or continuation of a certain treatment. However, communication of potential risks will remain demanding for the treating physicians.

## Figures and Tables

**Table 1:** Demographic characteristics of the study population.

Characteristic	Total SES *n* = 5,781	nAMD *n* = 3,631	DME *n* = 1,226	RVO *n* = 744
Gender				
Male	2,530 (43.8%)	1,401 (38.6%)	705 (57.5%)	353 (47.5%)
Female	3,235 (56.0%)	2,222 (61.2%)	515 (42.0%)	389 (52.3%)
Missing	16 (0.3%)	8 (0.2%)	6 (0.5%)	2 (0.3%)
Age (years)				
Mean (SD)	74.6 (10.3)	77.9 (8.1)	67.6 (10.8)	71.0 (10.9)
BMI (kg/m^2^)				
Mean (SD)	27.2 (4.5)	26.6 (4.0)	29.3 (5.2)	27.1 (4.3)
Total number of injections per patient (observational period)				
Mean (SD)	5.7 (3.8)	5.7 (3.7)	5.5 (3.6)	6.0 (4.1)

Due to low patient numbers for the mCNV population, a breakdown by demographic characteristics is not presented. BMI, body mass index; DME, diabetic macular edema; mCNV, myopic choroidal neovascularization; nAMD, neovascular age-related macular degeneration; RVO, retinal vein occlusion; SD, standard deviation; SES, safety evaluation set.

**Table 2:** Most commonly reported adverse events (MedDRA PT level), by category.

Category of patients with events (%)	nsAE	SAE	nsADR	sADR
	451 (100.0%)	417 (100.0%)	498 (100.0%)	213 (100.0%)
Most frequently reported events (% of patients with events in respective category)	Intraocular pressure increased (14.4%)	Visual acuity reduced (11.8%)	Drug ineffective (20.3%)	Visual acuity reduced (18.3%)
	Cataract operation (12.6%)	Fall (5.8%)	Visual acuity reduced (16.5%)	Death (11.3%)
	Visual acuity reduced (7.5%)	Pneumonia (5.3%)	Intraocular pressure increased (13.1%)	Cerebrovascular accident (7.5%)
	Posterior capsule opacification (6.0%)	Cardiac failure (5.0%)	Adverse event^*∗*^ (5.2%)	Intraocular pressure increased (4.2%)
	Conjunctivitis (5.5%)	Vitreous haemorrhage (4.6%)		Retinal haemorrhage (4.2%)
	Cataract (5.3%)	Death (4.6%)		Hospitalization (4.2%)
		Retinal haemorrhage (4.3%)		

^*∗*^Adverse event not further classified MedDRA, Medical Dictionary for Regulatory Activities; nsADR, nonserious adverse drug reaction; nsAE, nonserious adverse event; PT, preferred term; sADR, serious adverse drug reaction; SAE, serious adverse event.

**Table 3:** Most frequently reported adverse events (MedDRA PTs), by time point of occurrence.

All events MedDRA PT	Month of start of event							
	0–3	4–6	7–9	10–12	13–15	16–18	19–21	22 and later
	586 (100%)	481 (100%)	329 (100%)	289 (100%)	261 (100%)	217 (100%)	178 (100%)	243 (100%)
	*n* (%)	*n* (%)	*n* (%)	*n* (%)	*n* (%)	*n* (%)	*n* (%)	*n* (%)
Visual acuity reduced	15.5	6.2	8.5	5.5	5.7	2.8	4.5	1.6
Intraocular pressure increased	11.3	7.9	4.9	5.9	6.1	7.4	6.7	7.4
Drug ineffective	4.1	6.4	4.3	4.8	6.5	3.2	1.7	2.5
Cataract operation	2.2	4.6	4.6	1.4	2.3	1.4	3.4	2.5
Ocular hypertension	2.2	1.0	1.5	1.7	0.0	0.0	0.0	0.0
Retinal haemorrhage	0.7	1.9	2.7	2.1	2.3	1.4	1.7	1.2
Adverse event^*∗*^	0.7	2.5	2.1	1.7	0.0	0.0	0.0	0.0
Cataract	0.3	1.2	2.4	2.4	2.3	1.8	1.1	1.2
Fall	1.4	1.7	0.9	1.4	0.8	4.1	2.8	2.1
Posterior capsule opacification	0.9	1.0	1.2	2.8	3.4	1.4	1.1	2.1
Death	1.0	0.6	1.2	2.8	1.5	3.2	1.1	3.3
Macular oedema	0.9	1.7	1.5	1.0	1.1	1.4	1.1	0.4
Vitreous haemorrhage	1.4	1.2	1.2	1.0	2.3	0.9	1.1	2.1
Eye irritation	2.6	0.6	0.3	0.0	0.0	0.0	0.0	0.0
Nasopharyngitis	0.9	0.8	2.1	0.7	0.0	0.0	0.0	0.0
Pneumonia	0.7	1.2	0.6	2.1	1.1	1.4	1.7	0.0
Cerebrovascular accident	1.5	0.2	0.9	0.3	0.8	3.2	1.1	1.6
Cardiac failure	0.2	1.0	0.3	0.7	0.8	1.4	3.4	1.2
Conjunctivitis	1.0	0.6	1.2	0.7	1.1	0.9	2.8	1.6
General physical health deterioration	0.3	0.6	0.3	1.4	1.1	0.0	0.6	2.9
Glaucoma	0.3	0.4	0.9	1.0	0.8	0.5	0.6	2.1
Hospitalization	0.0	0.2	0.6	0.3	0.0	1.4	0.6	2.1
Macular scar	0.0	0.0	0.0	0.0	0.8	0.5	1.7	1.2

^*∗*^Adverse event not further classified. Table shows events with an incidence rate of ≥1% per year in total. Percentages were calculated based on the total number of events in respective time period. MedDRA, Medical Dictionary for Regulatory Activities; PT, preferred term.

**Table 4:** Cox proportional hazard regression for occurrence of any AE.

Parameter	Estimate	Standard error	*P* value	Hazard ratio
Sex	−0.091	0.063	0.147	0.913
Baseline visual acuity	−0.005	0.001	<0.001	0.995
Age	0.012	0.003	<0.001	1.012
BMI	0.013	0.007	0.076	1.013
Indication	0.215	0.043	<0.001	1.240
Physician location	−0.382	0.081	<0.001	0.682

AE, adverse event; BMI, body mass index.

## Data Availability

The safety data used to support the findings of this study have not been made available due to German data protection law. They may be made available from the corresponding author upon request. The participating OCEAN study sites are provided in the Supplementary Materials.

## Supplementary Materials

Supplementary Materials Figure S1: comparison of the reported incidence rates for selected ocular adverse events among the nAMD subpopulation in the OCEAN study and in the treat-and-extend group of the randomized controlled clinical study TREND [37]. All assessed ocular AEs were reported less frequently in OCEAN than in the TREND study. Percentages are based on number of patients in the respective study treatment group. IOP, intraocular pressure; nAMD, neovascular age-related macular degeneration. Figure S2: comparison of the reported incidence rates for selected ocular adverse events among the DME subpopulation in the OCEAN study and in the PRN group of the randomized controlled clinical study RETAIN [38]. Most assessed ocular AEs were reported less frequently in OCEAN than in the RETAIN study. Note that the incidence rate for retinal haemorrhage was 0.0% in RETAIN. Percentages are based on number of patients in the respective study treatment group. AE, adverse event; DME, diabetic macula edema; IOP, intraocular pressure. Figure S3: comparison of the reported incidence rates for selected ocular adverse events among the RVO subpopulation in the OCEAN study and in the CRYSTAL study [39]. Most assessed ocular AEs were reported less frequently in OCEAN than in the CRYSTAL study. Note that no incidence rate for retinal haemorrhage was published in CRYSTAL. Percentages are based on number of patients in the respective study treatment group. AE, adverse event; IOP, intraocular pressure; RVO, retinal vein occlusion. Supplementary Table 1. Patient-based adverse events–MedDRA primary SOCs and preferred terms (multiple responses)–SES.
