# Supplementary material for: Reporting of Safety Events during Anti-VEGF Treatment: Pharmacovigilance in a Noninterventional Trial
Source: J Ophthalmol. 2020 Oct 6;2020:8652370. doi: 10.1155/2020/8652370 (PMC7558801; doi:10.1155/2020/8652370)
Supplement: Supplementary Materials — Figure S1: comparison of the reported incidence rates for selected ocular adverse events among the nAMD subpopulation in the OCEAN study and in the treat-and-extend group of the randomized controlled clinical study TREND [37]. All assessed ocular AEs were reported less frequently in OCEAN than in the TREND study. Percentages are based on number of patients in the respective study treatment group. IOP, intraocular pressure; nAMD, neovascular age-related macular degeneration. Figure S2: comparison of the reported incidence rates for selected ocular adverse events among the DME subpopulation in the OCEAN study and in the PRN group of the randomized controlled clinical study RETAIN [38]. Most assessed ocular AEs were reported less frequently in OCEAN than in the RETAIN study. Note that the incidence rate for retinal haemorrhage was 0.0% in RETAIN. Percentages are based on number of patients in the respective study treatment group. AE, adverse event; DME, diabetic macula edema; IOP, intraocular pressure. Figure S3: comparison of the reported incidence rates for selected ocular adverse events among the RVO subpopulation in the OCEAN study and in the CRYSTAL study [39]. Most assessed ocular AEs were reported less frequently in OCEAN than in the CRYSTAL study. Note that no incidence rate for retinal haemorrhage was published in CRYSTAL. Percentages are based on number of patients in the respective study treatment group. AE, adverse event; IOP, intraocular pressure; RVO, retinal vein occlusion. Supplementary Table 1. Patient-based adverse events–MedDRA primary SOCs and preferred terms (multiple responses)–SES. [file 8652370.f1.docx]

**Supplementary Figures**


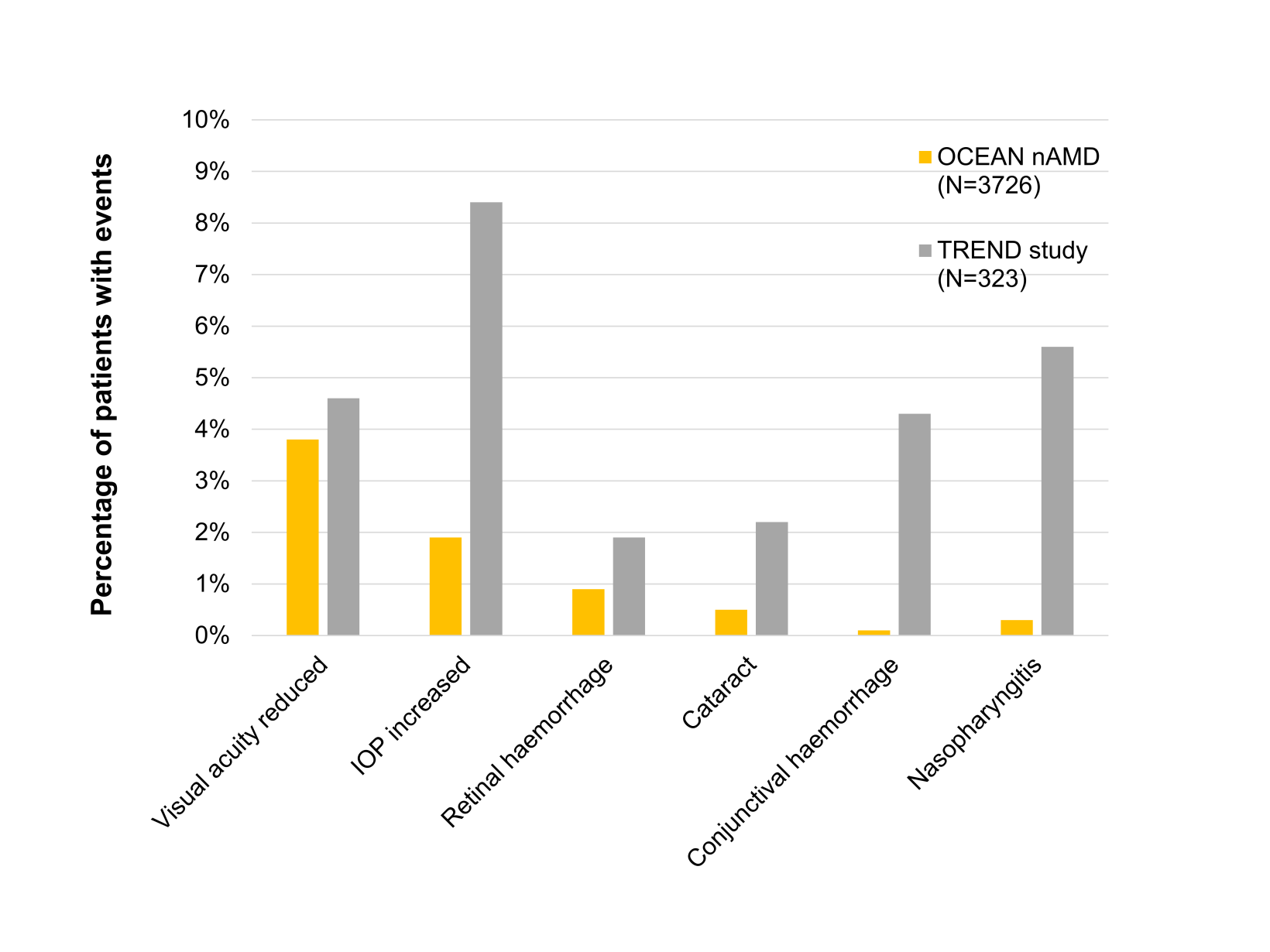


Figure S1: Comparison of the reported incidence rates for selected ocular adverse events among the nAMD subpopulation in OCEAN study and in the Treat & Extend group of the randomized controlled clinical study TREND [38].

All assessed ocular AEs were reported less frequently in OCEAN than in the TREND study. Percentages are based on number of patients in respective study treatment group. IOP: intraocular pressure, nAMD: neovascular age-related macular degeneration.


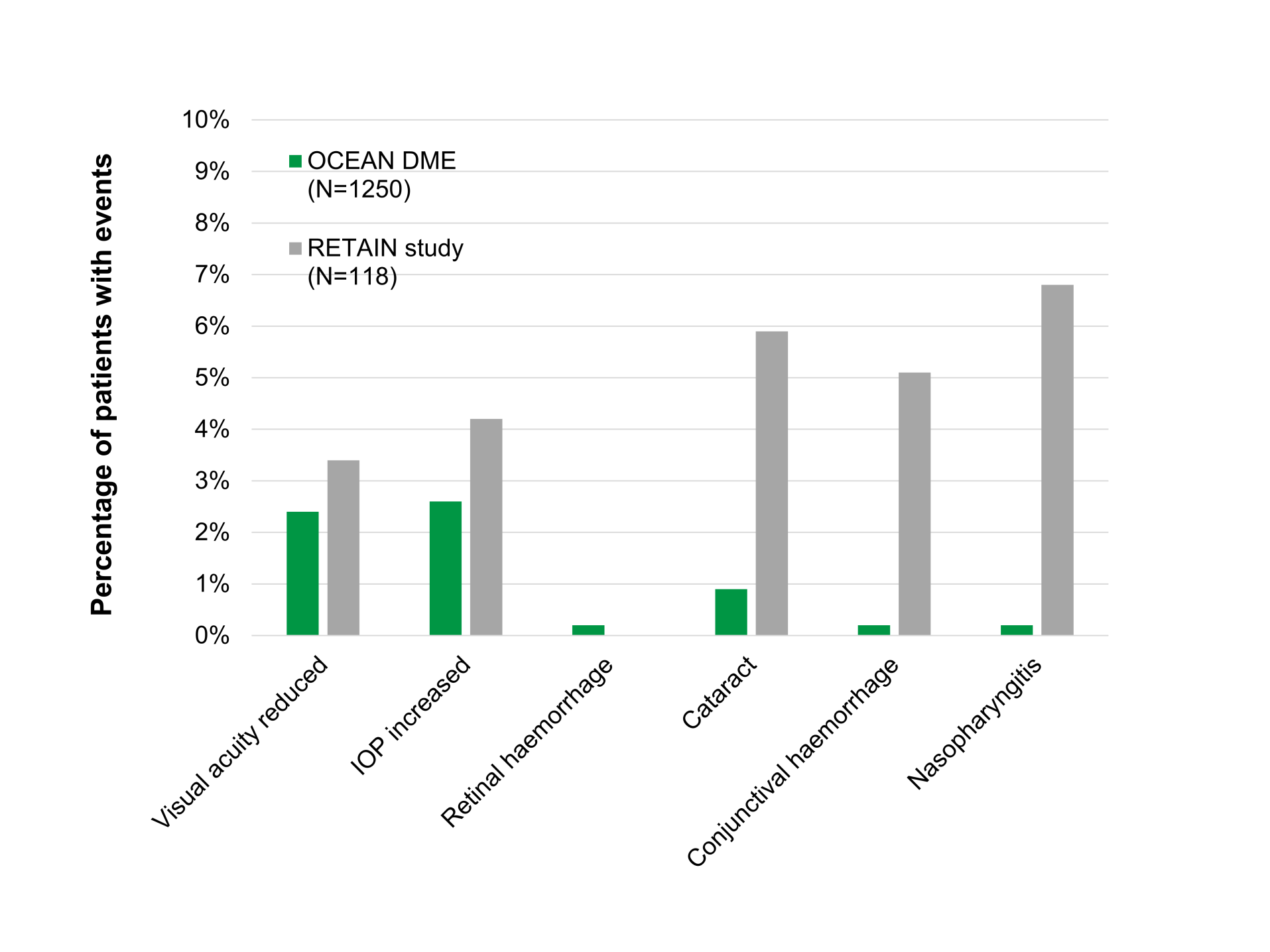


Figure S2: Comparison of the reported incidence rates for selected ocular adverse events among the DME subpopulation in OCEAN study and in the PRN group of the randomized controlled clinical study RETAIN [39].

Most assessed ocular AEs were reported less frequently in OCEAN than in the RETAIN study. Note that the incidence rate for retinal haemorrhage was 0.0% in RETAIN. Percentages are based on number of patients in respective study treatment group.

AE: adverse event, DME: diabetic macula edema, IOP: intraocular pressure.


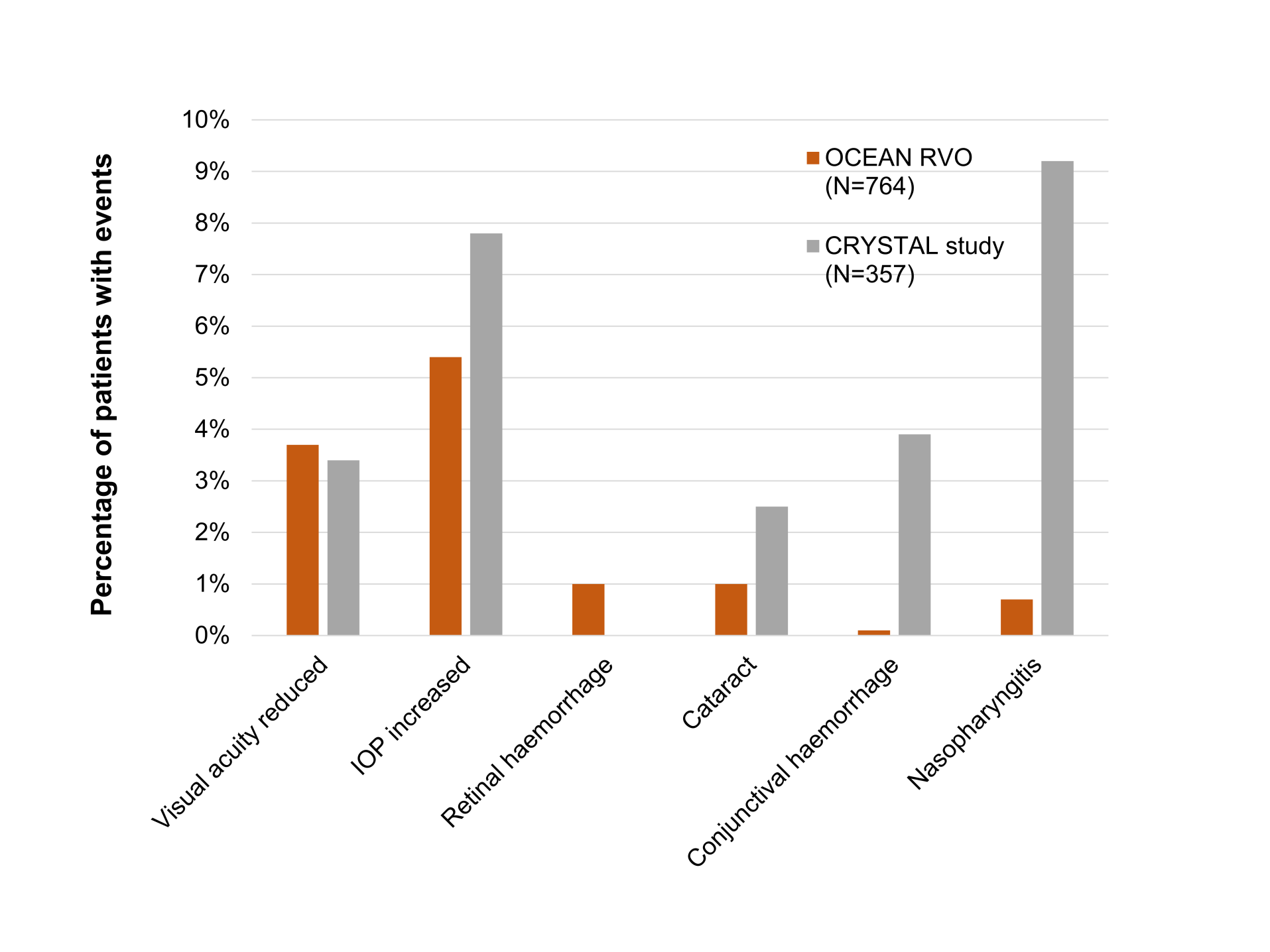


Figure S3: Comparison of the reported incidence rates for selected ocular adverse events among the RVO subpopulation in OCEAN study and in the CRYSTAL study [40].

Most assessed ocular AEs were reported less frequently in OCEAN than in the CRYSTAL study. Note that no incidence rate for retinal haemorrhage was published in CRYSTAL. Percentages are based on number of patients in respective study treatment group.

AE: adverse event, IOP: intraocular pressure, RVO: retinal vein occlusion.

**Supplementary Table 1.** Patient-based Adverse Events – MedDRA primary SOCs and Preferred Terms (multiple responses) - SES

|  |  | Number of patients | | | | |
| --- | --- | --- | --- | --- | --- | --- |
|  |  | Total | nsAE | SAE | nsADR | SADR |
|  |  | N (%) | N (%) | N (%) | N (%) | N (%) |
| Patients SAF |  | 5781 ( 100.0%) | 451 ( 100.0%) | 417 ( 100.0%) | 498 ( 100.0%) | 213 ( 100.0%) |
|  |  |  |  |  |  |  |
| Eye disorders | All Patients | 543 ( 9.4%) | 193 ( 42.8%) | 148 ( 35.5%) | 204 ( 41.0%) | 105 ( 49.3%) |
|  | Visual acuity reduced | 201 ( 3.5%) | 34 ( 7.5%) | 49 ( 11.8%) | 82 ( 16.5%) | 39 ( 18.3%) |
|  | Retinal haemorrhage | 43 ( 0.7%) | 7 ( 1.6%) | 18 ( 4.3%) | 9 ( 1.8%) | 9 ( 4.2%) |
|  | Posterior capsule opacification | 42 ( 0.7%) | 27 ( 6.0%) | 5 ( 1.2%) | 10 ( 2.0%) | 1 ( 0.5%) |
|  | Cataract | 38 ( 0.7%) | 24 ( 5.3%) | 9 ( 2.2%) | 2 ( 0.4%) | 3 ( 1.4%) |
|  | Vitreous haemorrhage | 31 ( 0.5%) | 4 ( 0.9%) | 19 ( 4.6%) | 2 ( 0.4%) | 6 ( 2.8%) |
|  | Macular oedema | 30 ( 0.5%) | 13 ( 2.9%) | 6 ( 1.4%) | 8 ( 1.6%) | 3 ( 1.4%) |
|  | Retinal oedema | 26 ( 0.4%) | 5 ( 1.1%) | 2 ( 0.5%) | 19 ( 3.8%) | 0 ( 0.00%) |
|  | Eye irritation | 21 ( 0.4%) | 6 ( 1.3%) | 0 ( 0.00%) | 15 ( 3.0%) | 0 ( 0.00%) |
|  | Glaucoma | 19 ( 0.3%) | 4 ( 0.9%) | 8 ( 1.9%) | 1 ( 0.2%) | 6 ( 2.8%) |
|  | Retinal cyst | 18 ( 0.3%) | 5 ( 1.1%) | 1 ( 0.2%) | 10 ( 2.0%) | 2 ( 0.9%) |
|  | Ocular hypertension | 17 ( 0.3%) | 9 ( 2.0%) | 3 ( 0.7%) | 3 ( 0.6%) | 3 ( 1.4%) |
|  | Retinal disorder | 17 ( 0.3%) | 3 ( 0.7%) | 0 ( 0.00%) | 13 ( 2.6%) | 1 ( 0.5%) |
|  | Subretinal fluid | 16 ( 0.3%) | 3 ( 0.7%) | 1 ( 0.2%) | 8 ( 1.6%) | 4 ( 1.9%) |
|  | Neovascular age-related macular degeneration | 15 ( 0.3%) | 3 ( 0.7%) | 5 ( 1.2%) | 4 ( 0.8%) | 4 ( 1.9%) |
|  | Detachment of retinal pigment epithelium | 13 ( 0.2%) | 0 ( 0.00%) | 5 ( 1.2%) | 1 ( 0.2%) | 7 ( 3.3%) |
|  | Macular scar | 13 ( 0.2%) | 1 ( 0.2%) | 7 ( 1.7%) | 1 ( 0.2%) | 4 ( 1.9%) |
|  | Retinal vein occlusion | 13 ( 0.2%) | 1 ( 0.2%) | 6 ( 1.4%) | 0 ( 0.00%) | 6 ( 2.8%) |
|  | Retinal degeneration | 12 ( 0.2%) | 1 ( 0.2%) | 4 ( 1.0%) | 1 ( 0.2%) | 6 ( 2.8%) |
|  | Eye pain | 11 ( 0.2%) | 2 ( 0.4%) | 1 ( 0.2%) | 5 ( 1.0%) | 3 ( 1.4%) |
|  | Metamorphopsia | 11 ( 0.2%) | 0 ( 0.00%) | 1 ( 0.2%) | 10 ( 2.0%) | 0 ( 0.00%) |
|  | Retinal scar | 11 ( 0.2%) | 0 ( 0.00%) | 4 ( 1.0%) | 4 ( 0.8%) | 3 ( 1.4%) |
|  | Corneal erosion | 10 ( 0.2%) | 3 ( 0.7%) | 0 ( 0.00%) | 6 ( 1.2%) | 1 ( 0.5%) |
|  | Retinal ischaemia | 10 ( 0.2%) | 1 ( 0.2%) | 4 ( 1.0%) | 2 ( 0.4%) | 3 ( 1.4%) |
|  | Choroidal neovascularisation | 9 ( 0.2%) | 3 ( 0.7%) | 3 ( 0.7%) | 2 ( 0.4%) | 1 ( 0.5%) |
|  | Conjunctival haemorrhage | 9 ( 0.2%) | 3 ( 0.7%) | 0 ( 0.00%) | 5 ( 1.0%) | 1 ( 0.5%) |
|  | Ocular hyperaemia | 9 ( 0.2%) | 5 ( 1.1%) | 0 ( 0.00%) | 2 ( 0.4%) | 2 ( 0.9%) |
|  | Retinal detachment | 9 ( 0.2%) | 1 ( 0.2%) | 5 ( 1.2%) | 0 ( 0.00%) | 3 ( 1.4%) |
|  | Retinal pigment epithelial tear | 9 ( 0.2%) | 0 ( 0.00%) | 4 ( 1.0%) | 0 ( 0.00%) | 5 ( 2.3%) |
|  | Diabetic retinopathy | 8 ( 0.1%) | 3 ( 0.7%) | 3 ( 0.7%) | 1 ( 0.2%) | 1 ( 0.5%) |
|  | Vision blurred | 8 ( 0.1%) | 1 ( 0.2%) | 1 ( 0.2%) | 4 ( 0.8%) | 2 ( 0.9%) |
|  | Conjunctival irritation | 7 ( 0.1%) | 1 ( 0.2%) | 0 ( 0.00%) | 6 ( 1.2%) | 0 ( 0.00%) |
|  | Visual impairment | 7 ( 0.1%) | 2 ( 0.4%) | 0 ( 0.00%) | 5 ( 1.0%) | 0 ( 0.00%) |
|  | Open angle glaucoma | 6 ( 0.1%) | 1 ( 0.2%) | 2 ( 0.5%) | 2 ( 0.4%) | 1 ( 0.5%) |
|  | Subretinal fibrosis | 6 ( 0.1%) | 0 ( 0.00%) | 3 ( 0.7%) | 0 ( 0.00%) | 3 ( 1.4%) |
|  | Age-related macular degeneration | 5 ( 0.1%) | 0 ( 0.00%) | 1 ( 0.2%) | 1 ( 0.2%) | 3 ( 1.4%) |
|  | Blindness | 5 ( 0.1%) | 0 ( 0.00%) | 1 ( 0.2%) | 0 ( 0.00%) | 4 ( 1.9%) |
|  | Dry eye | 5 ( 0.1%) | 4 ( 0.9%) | 0 ( 0.00%) | 1 ( 0.2%) | 0 ( 0.00%) |
|  | Lacrimation increased | 5 ( 0.1%) | 3 ( 0.7%) | 0 ( 0.00%) | 2 ( 0.4%) | 0 ( 0.00%) |
|  | Macular fibrosis | 5 ( 0.1%) | 2 ( 0.4%) | 2 ( 0.5%) | 0 ( 0.00%) | 1 ( 0.5%) |
|  | Retinopathy proliferative | 5 ( 0.1%) | 1 ( 0.2%) | 2 ( 0.5%) | 1 ( 0.2%) | 1 ( 0.5%) |
|  | Blepharitis | 4 ( 0.1%) | 4 ( 0.9%) | 0 ( 0.00%) | 0 ( 0.00%) | 0 ( 0.00%) |
|  | Cystoid macular oedema | 4 ( 0.1%) | 2 ( 0.4%) | 0 ( 0.00%) | 1 ( 0.2%) | 1 ( 0.5%) |
|  | Eye disorder | 4 ( 0.1%) | 2 ( 0.4%) | 2 ( 0.5%) | 1 ( 0.2%) | 0 ( 0.00%) |
|  | Macular degeneration | 4 ( 0.1%) | 1 ( 0.2%) | 2 ( 0.5%) | 1 ( 0.2%) | 0 ( 0.00%) |
|  | Retinal aneurysm | 4 ( 0.1%) | 1 ( 0.2%) | 1 ( 0.2%) | 1 ( 0.2%) | 1 ( 0.5%) |
|  | Retinal exudates | 4 ( 0.1%) | 3 ( 0.7%) | 0 ( 0.00%) | 1 ( 0.2%) | 0 ( 0.00%) |
|  | Retinal vein thrombosis | 4 ( 0.1%) | 1 ( 0.2%) | 3 ( 0.7%) | 0 ( 0.00%) | 0 ( 0.00%) |
|  | Vitreous adhesions | 4 ( 0.1%) | 2 ( 0.4%) | 1 ( 0.2%) | 1 ( 0.2%) | 0 ( 0.00%) |
|  | Eye haemorrhage | 3 ( 0.1%) | 0 ( 0.00%) | 2 ( 0.5%) | 0 ( 0.00%) | 1 ( 0.5%) |
|  | Macular cyst | 3 ( 0.1%) | 1 ( 0.2%) | 2 ( 0.5%) | 0 ( 0.00%) | 0 ( 0.00%) |
|  | Macular hole | 3 ( 0.1%) | 0 ( 0.00%) | 2 ( 0.5%) | 0 ( 0.00%) | 1 ( 0.5%) |
|  | Maculopathy | 3 ( 0.1%) | 1 ( 0.2%) | 1 ( 0.2%) | 1 ( 0.2%) | 0 ( 0.00%) |
|  | Ocular discomfort | 3 ( 0.1%) | 2 ( 0.4%) | 0 ( 0.00%) | 1 ( 0.2%) | 0 ( 0.00%) |
|  | Punctate keratitis | 3 ( 0.1%) | 2 ( 0.4%) | 0 ( 0.00%) | 1 ( 0.2%) | 0 ( 0.00%) |
|  | Vitreous detachment | 3 ( 0.1%) | 0 ( 0.00%) | 1 ( 0.2%) | 1 ( 0.2%) | 1 ( 0.5%) |
|  | Vitreous disorder | 3 ( 0.1%) | 1 ( 0.2%) | 0 ( 0.00%) | 2 ( 0.4%) | 0 ( 0.00%) |
|  | Vitreous floaters | 3 ( 0.1%) | 0 ( 0.00%) | 0 ( 0.00%) | 3 ( 0.6%) | 0 ( 0.00%) |
|  | Abnormal sensation in eye | 2 ( 0.0%) | 2 ( 0.4%) | 0 ( 0.00%) | 0 ( 0.00%) | 0 ( 0.00%) |
|  | Anterior chamber cell | 2 ( 0.0%) | 0 ( 0.00%) | 0 ( 0.00%) | 1 ( 0.2%) | 1 ( 0.5%) |
|  | Anterior chamber disorder | 2 ( 0.0%) | 0 ( 0.00%) | 0 ( 0.00%) | 2 ( 0.4%) | 0 ( 0.00%) |
|  | Conjunctival oedema | 2 ( 0.0%) | 1 ( 0.2%) | 0 ( 0.00%) | 1 ( 0.2%) | 0 ( 0.00%) |
|  | Conjunctivitis allergic | 2 ( 0.0%) | 1 ( 0.2%) | 0 ( 0.00%) | 1 ( 0.2%) | 0 ( 0.00%) |
|  | Diabetic retinal oedema | 2 ( 0.0%) | 0 ( 0.00%) | 1 ( 0.2%) | 0 ( 0.00%) | 1 ( 0.5%) |
|  | Diplopia | 2 ( 0.0%) | 0 ( 0.00%) | 2 ( 0.5%) | 0 ( 0.00%) | 0 ( 0.00%) |
|  | Eye allergy | 2 ( 0.0%) | 2 ( 0.4%) | 0 ( 0.00%) | 0 ( 0.00%) | 0 ( 0.00%) |
|  | Eye pruritus | 2 ( 0.0%) | 2 ( 0.4%) | 0 ( 0.00%) | 0 ( 0.00%) | 0 ( 0.00%) |
|  | Eyelid oedema | 2 ( 0.0%) | 0 ( 0.00%) | 0 ( 0.00%) | 2 ( 0.4%) | 0 ( 0.00%) |
|  | Foreign body sensation in eyes | 2 ( 0.0%) | 1 ( 0.2%) | 0 ( 0.00%) | 1 ( 0.2%) | 0 ( 0.00%) |
|  | Iritis | 2 ( 0.0%) | 2 ( 0.4%) | 0 ( 0.00%) | 0 ( 0.00%) | 0 ( 0.00%) |
|  | Macular ischaemia | 2 ( 0.0%) | 0 ( 0.00%) | 1 ( 0.2%) | 0 ( 0.00%) | 1 ( 0.5%) |
|  | Ophthalmoplegia | 2 ( 0.0%) | 0 ( 0.00%) | 1 ( 0.2%) | 0 ( 0.00%) | 1 ( 0.5%) |
|  | Optic atrophy | 2 ( 0.0%) | 0 ( 0.00%) | 1 ( 0.2%) | 0 ( 0.00%) | 1 ( 0.5%) |
|  | Optic disc disorder | 2 ( 0.0%) | 0 ( 0.00%) | 2 ( 0.5%) | 0 ( 0.00%) | 0 ( 0.00%) |
|  | Photopsia | 2 ( 0.0%) | 1 ( 0.2%) | 0 ( 0.00%) | 1 ( 0.2%) | 0 ( 0.00%) |
|  | Retinal artery occlusion | 2 ( 0.0%) | 0 ( 0.00%) | 0 ( 0.00%) | 0 ( 0.00%) | 2 ( 0.9%) |
|  | Retinal depigmentation | 2 ( 0.0%) | 0 ( 0.00%) | 2 ( 0.5%) | 0 ( 0.00%) | 0 ( 0.00%) |
|  | Retinal fibrosis | 2 ( 0.0%) | 0 ( 0.00%) | 0 ( 0.00%) | 1 ( 0.2%) | 1 ( 0.5%) |
|  | Retinal neovascularisation | 2 ( 0.0%) | 0 ( 0.00%) | 2 ( 0.5%) | 0 ( 0.00%) | 0 ( 0.00%) |
|  | Vitritis | 2 ( 0.0%) | 1 ( 0.2%) | 0 ( 0.00%) | 0 ( 0.00%) | 1 ( 0.5%) |
|  | Amaurosis | 1 ( 0.0%) | 0 ( 0.00%) | 0 ( 0.00%) | 0 ( 0.00%) | 1 ( 0.5%) |
|  | Amaurosis fugax | 1 ( 0.0%) | 0 ( 0.00%) | 0 ( 0.00%) | 0 ( 0.00%) | 1 ( 0.5%) |
|  | Angle closure glaucoma | 1 ( 0.0%) | 0 ( 0.00%) | 1 ( 0.2%) | 0 ( 0.00%) | 0 ( 0.00%) |
|  | Anterior capsule contraction | 1 ( 0.0%) | 1 ( 0.2%) | 0 ( 0.00%) | 0 ( 0.00%) | 0 ( 0.00%) |
|  | Blindness unilateral | 1 ( 0.0%) | 0 ( 0.00%) | 0 ( 0.00%) | 0 ( 0.00%) | 1 ( 0.5%) |
|  | Chalazion | 1 ( 0.0%) | 1 ( 0.2%) | 0 ( 0.00%) | 0 ( 0.00%) | 0 ( 0.00%) |
|  | Choroidal detachment | 1 ( 0.0%) | 0 ( 0.00%) | 1 ( 0.2%) | 0 ( 0.00%) | 0 ( 0.00%) |
|  | Choroidal haemorrhage | 1 ( 0.0%) | 1 ( 0.2%) | 0 ( 0.00%) | 0 ( 0.00%) | 0 ( 0.00%) |
|  | Conjunctival erosion | 1 ( 0.0%) | 1 ( 0.2%) | 0 ( 0.00%) | 0 ( 0.00%) | 0 ( 0.00%) |
|  | Conjunctival hyperaemia | 1 ( 0.0%) | 1 ( 0.2%) | 0 ( 0.00%) | 0 ( 0.00%) | 0 ( 0.00%) |
|  | Corneal decompensation | 1 ( 0.0%) | 0 ( 0.00%) | 0 ( 0.00%) | 0 ( 0.00%) | 1 ( 0.5%) |
|  | Corneal oedema | 1 ( 0.0%) | 1 ( 0.2%) | 0 ( 0.00%) | 0 ( 0.00%) | 0 ( 0.00%) |
|  | Dry age-related macular degeneration | 1 ( 0.0%) | 0 ( 0.00%) | 1 ( 0.2%) | 0 ( 0.00%) | 0 ( 0.00%) |
|  | Ectropion | 1 ( 0.0%) | 1 ( 0.2%) | 0 ( 0.00%) | 0 ( 0.00%) | 0 ( 0.00%) |
|  | Entropion | 1 ( 0.0%) | 1 ( 0.2%) | 0 ( 0.00%) | 0 ( 0.00%) | 0 ( 0.00%) |
|  | Episcleritis | 1 ( 0.0%) | 1 ( 0.2%) | 0 ( 0.00%) | 0 ( 0.00%) | 0 ( 0.00%) |
|  | Erythema of eyelid | 1 ( 0.0%) | 1 ( 0.2%) | 0 ( 0.00%) | 0 ( 0.00%) | 0 ( 0.00%) |
|  | Eye oedema | 1 ( 0.0%) | 0 ( 0.00%) | 0 ( 0.00%) | 1 ( 0.2%) | 0 ( 0.00%) |
|  | Eyelid haematoma | 1 ( 0.0%) | 1 ( 0.2%) | 0 ( 0.00%) | 0 ( 0.00%) | 0 ( 0.00%) |
|  | Eyelid margin crusting | 1 ( 0.0%) | 1 ( 0.2%) | 0 ( 0.00%) | 0 ( 0.00%) | 0 ( 0.00%) |
|  | Eyelid pain | 1 ( 0.0%) | 1 ( 0.2%) | 0 ( 0.00%) | 0 ( 0.00%) | 0 ( 0.00%) |
|  | Eyelid thickening | 1 ( 0.0%) | 1 ( 0.2%) | 0 ( 0.00%) | 0 ( 0.00%) | 0 ( 0.00%) |
|  | Hyphaema | 1 ( 0.0%) | 0 ( 0.00%) | 1 ( 0.2%) | 0 ( 0.00%) | 0 ( 0.00%) |
|  | Hypotony of eye | 1 ( 0.0%) | 0 ( 0.00%) | 1 ( 0.2%) | 0 ( 0.00%) | 0 ( 0.00%) |
|  | Iris haemorrhage | 1 ( 0.0%) | 0 ( 0.00%) | 0 ( 0.00%) | 0 ( 0.00%) | 1 ( 0.5%) |
|  | Iris neovascularisation | 1 ( 0.0%) | 0 ( 0.00%) | 1 ( 0.2%) | 0 ( 0.00%) | 0 ( 0.00%) |
|  | Lagophthalmos | 1 ( 0.0%) | 0 ( 0.00%) | 0 ( 0.00%) | 1 ( 0.2%) | 0 ( 0.00%) |
|  | Lens disorder | 1 ( 0.0%) | 0 ( 0.00%) | 0 ( 0.00%) | 0 ( 0.00%) | 1 ( 0.5%) |
|  | Lenticular opacities | 1 ( 0.0%) | 0 ( 0.00%) | 0 ( 0.00%) | 0 ( 0.00%) | 1 ( 0.5%) |
|  | Narrow anterior chamber angle | 1 ( 0.0%) | 0 ( 0.00%) | 0 ( 0.00%) | 0 ( 0.00%) | 1 ( 0.5%) |
|  | Non-infectious endophthalmitis | 1 ( 0.0%) | 0 ( 0.00%) | 0 ( 0.00%) | 0 ( 0.00%) | 1 ( 0.5%) |
|  | Normal tension glaucoma | 1 ( 0.0%) | 0 ( 0.00%) | 1 ( 0.2%) | 0 ( 0.00%) | 0 ( 0.00%) |
|  | Ocular ischaemic syndrome | 1 ( 0.0%) | 0 ( 0.00%) | 1 ( 0.2%) | 0 ( 0.00%) | 0 ( 0.00%) |
|  | Optic nerve cupping | 1 ( 0.0%) | 0 ( 0.00%) | 1 ( 0.2%) | 0 ( 0.00%) | 0 ( 0.00%) |
|  | Retinal pigment epitheliopathy | 1 ( 0.0%) | 1 ( 0.2%) | 0 ( 0.00%) | 0 ( 0.00%) | 0 ( 0.00%) |
|  | Retinal pigmentation | 1 ( 0.0%) | 0 ( 0.00%) | 1 ( 0.2%) | 0 ( 0.00%) | 0 ( 0.00%) |
|  | Retinal tear | 1 ( 0.0%) | 0 ( 0.00%) | 1 ( 0.2%) | 0 ( 0.00%) | 0 ( 0.00%) |
|  | Retinal telangiectasia | 1 ( 0.0%) | 0 ( 0.00%) | 1 ( 0.2%) | 0 ( 0.00%) | 0 ( 0.00%) |
|  | Retinal thickening | 1 ( 0.0%) | 0 ( 0.00%) | 1 ( 0.2%) | 0 ( 0.00%) | 0 ( 0.00%) |
|  | Retinal vasculitis | 1 ( 0.0%) | 1 ( 0.2%) | 0 ( 0.00%) | 0 ( 0.00%) | 0 ( 0.00%) |
|  | Strabismus | 1 ( 0.0%) | 0 ( 0.00%) | 0 ( 0.00%) | 0 ( 0.00%) | 1 ( 0.5%) |
|  | Trichiasis | 1 ( 0.0%) | 1 ( 0.2%) | 0 ( 0.00%) | 0 ( 0.00%) | 0 ( 0.00%) |
|  | Ulcerative keratitis | 1 ( 0.0%) | 0 ( 0.00%) | 0 ( 0.00%) | 1 ( 0.2%) | 0 ( 0.00%) |
|  | Uveitis | 1 ( 0.0%) | 0 ( 0.00%) | 1 ( 0.2%) | 0 ( 0.00%) | 0 ( 0.00%) |
|  |  |  |  |  |  |  |
| General disorders and administration site conditions | All Patients | 336 ( 5.8%) | 52 ( 11.5%) | 63 ( 15.1%) | 192 ( 38.6%) | 43 ( 20.2%) |
|  | Drug ineffective | 119 ( 2.1%) | 13 ( 2.9%) | 4 ( 1.0%) | 101 ( 20.3%) | 2 ( 0.9%) |
|  | Death | 43 ( 0.7%) | 0 ( 0.00%) | 19 ( 4.6%) | 0 ( 0.00%) | 24 ( 11.3%) |
|  | Adverse event | 31 ( 0.5%) | 4 ( 0.9%) | 0 ( 0.00%) | 26 ( 5.2%) | 1 ( 0.5%) |
|  | General physical health deterioration | 21 ( 0.4%) | 4 ( 0.9%) | 6 ( 1.4%) | 10 ( 2.0%) | 1 ( 0.5%) |
|  | Malaise | 17 ( 0.3%) | 3 ( 0.7%) | 1 ( 0.2%) | 13 ( 2.6%) | 0 ( 0.00%) |
|  | Condition aggravated | 15 ( 0.3%) | 6 ( 1.3%) | 3 ( 0.7%) | 4 ( 0.8%) | 2 ( 0.9%) |
|  | Therapy non-responder | 13 ( 0.2%) | 2 ( 0.4%) | 2 ( 0.5%) | 9 ( 1.8%) | 0 ( 0.00%) |
|  | Ill-defined disorder | 10 ( 0.2%) | 2 ( 0.4%) | 0 ( 0.00%) | 8 ( 1.6%) | 0 ( 0.00%) |
|  | Gait disturbance | 7 ( 0.1%) | 2 ( 0.4%) | 3 ( 0.7%) | 1 ( 0.2%) | 1 ( 0.5%) |
|  | Asthenia | 6 ( 0.1%) | 1 ( 0.2%) | 2 ( 0.5%) | 2 ( 0.4%) | 1 ( 0.5%) |
|  | Chest discomfort | 6 ( 0.1%) | 0 ( 0.00%) | 2 ( 0.5%) | 1 ( 0.2%) | 3 ( 1.4%) |
|  | Disease recurrence | 6 ( 0.1%) | 1 ( 0.2%) | 2 ( 0.5%) | 1 ( 0.2%) | 2 ( 0.9%) |
|  | Drug intolerance | 6 ( 0.1%) | 3 ( 0.7%) | 0 ( 0.00%) | 3 ( 0.6%) | 0 ( 0.00%) |
|  | Pain | 6 ( 0.1%) | 0 ( 0.00%) | 5 ( 1.2%) | 1 ( 0.2%) | 0 ( 0.00%) |
|  | Chest pain | 5 ( 0.1%) | 0 ( 0.00%) | 3 ( 0.7%) | 0 ( 0.00%) | 2 ( 0.9%) |
|  | Tachyphylaxis | 5 ( 0.1%) | 0 ( 0.00%) | 0 ( 0.00%) | 5 ( 1.0%) | 0 ( 0.00%) |
|  | Injection site pain | 4 ( 0.1%) | 3 ( 0.7%) | 0 ( 0.00%) | 1 ( 0.2%) | 0 ( 0.00%) |
|  | Oedema | 4 ( 0.1%) | 1 ( 0.2%) | 2 ( 0.5%) | 1 ( 0.2%) | 0 ( 0.00%) |
|  | Concomitant disease aggravated | 3 ( 0.1%) | 0 ( 0.00%) | 0 ( 0.00%) | 2 ( 0.4%) | 1 ( 0.5%) |
|  | Disease progression | 3 ( 0.1%) | 0 ( 0.00%) | 1 ( 0.2%) | 0 ( 0.00%) | 2 ( 0.9%) |
|  | Fatigue | 3 ( 0.1%) | 0 ( 0.00%) | 1 ( 0.2%) | 0 ( 0.00%) | 2 ( 0.9%) |
|  | Multiple organ dysfunction syndrome | 3 ( 0.1%) | 0 ( 0.00%) | 3 ( 0.7%) | 0 ( 0.00%) | 0 ( 0.00%) |
|  | Oedema peripheral | 3 ( 0.1%) | 0 ( 0.00%) | 3 ( 0.7%) | 0 ( 0.00%) | 0 ( 0.00%) |
|  | Therapeutic response decreased | 3 ( 0.1%) | 0 ( 0.00%) | 1 ( 0.2%) | 2 ( 0.4%) | 0 ( 0.00%) |
|  | Peripheral swelling | 2 ( 0.0%) | 2 ( 0.4%) | 0 ( 0.00%) | 0 ( 0.00%) | 0 ( 0.00%) |
|  | Therapeutic response delayed | 2 ( 0.0%) | 1 ( 0.2%) | 0 ( 0.00%) | 1 ( 0.2%) | 0 ( 0.00%) |
|  | Application site haemorrhage | 1 ( 0.0%) | 1 ( 0.2%) | 0 ( 0.00%) | 0 ( 0.00%) | 0 ( 0.00%) |
|  | Complication associated with device | 1 ( 0.0%) | 0 ( 0.00%) | 1 ( 0.2%) | 0 ( 0.00%) | 0 ( 0.00%) |
|  | Discomfort | 1 ( 0.0%) | 0 ( 0.00%) | 1 ( 0.2%) | 0 ( 0.00%) | 0 ( 0.00%) |
|  | Drug effect incomplete | 1 ( 0.0%) | 0 ( 0.00%) | 0 ( 0.00%) | 1 ( 0.2%) | 0 ( 0.00%) |
|  | Fibrosis | 1 ( 0.0%) | 0 ( 0.00%) | 0 ( 0.00%) | 0 ( 0.00%) | 1 ( 0.5%) |
|  | Foreign body reaction | 1 ( 0.0%) | 1 ( 0.2%) | 0 ( 0.00%) | 0 ( 0.00%) | 0 ( 0.00%) |
|  | Impaired healing | 1 ( 0.0%) | 0 ( 0.00%) | 1 ( 0.2%) | 0 ( 0.00%) | 0 ( 0.00%) |
|  | Inflammation | 1 ( 0.0%) | 1 ( 0.2%) | 0 ( 0.00%) | 0 ( 0.00%) | 0 ( 0.00%) |
|  | Influenza like illness | 1 ( 0.0%) | 1 ( 0.2%) | 0 ( 0.00%) | 0 ( 0.00%) | 0 ( 0.00%) |
|  | Injection site irritation | 1 ( 0.0%) | 0 ( 0.00%) | 0 ( 0.00%) | 1 ( 0.2%) | 0 ( 0.00%) |
|  | Injection site reaction | 1 ( 0.0%) | 0 ( 0.00%) | 0 ( 0.00%) | 1 ( 0.2%) | 0 ( 0.00%) |
|  | Necrosis | 1 ( 0.0%) | 1 ( 0.2%) | 0 ( 0.00%) | 0 ( 0.00%) | 0 ( 0.00%) |
|  | No adverse event | 1 ( 0.0%) | 0 ( 0.00%) | 1 ( 0.2%) | 0 ( 0.00%) | 0 ( 0.00%) |
|  | Polyp | 1 ( 0.0%) | 0 ( 0.00%) | 1 ( 0.2%) | 0 ( 0.00%) | 0 ( 0.00%) |
|  | Pyrexia | 1 ( 0.0%) | 0 ( 0.00%) | 1 ( 0.2%) | 0 ( 0.00%) | 0 ( 0.00%) |
|  | Sensation of foreign body | 1 ( 0.0%) | 1 ( 0.2%) | 0 ( 0.00%) | 0 ( 0.00%) | 0 ( 0.00%) |
|  | Swelling | 1 ( 0.0%) | 0 ( 0.00%) | 0 ( 0.00%) | 0 ( 0.00%) | 1 ( 0.5%) |
|  | Ulcer | 1 ( 0.0%) | 0 ( 0.00%) | 1 ( 0.2%) | 0 ( 0.00%) | 0 ( 0.00%) |
|  |  |  |  |  |  |  |
| Investigations | All Patients | 181 ( 3.1%) | 73 ( 16.2%) | 27 ( 6.5%) | 79 ( 15.9%) | 13 ( 6.1%) |
|  | Intraocular pressure increased | 144 ( 2.5%) | 65 ( 14.4%) | 14 ( 3.4%) | 65 ( 13.1%) | 9 ( 4.2%) |
|  | Angiogram abnormal | 5 ( 0.1%) | 0 ( 0.00%) | 1 ( 0.2%) | 4 ( 0.8%) | 0 ( 0.00%) |
|  | Intraocular pressure decreased | 5 ( 0.1%) | 0 ( 0.00%) | 0 ( 0.00%) | 5 ( 1.0%) | 0 ( 0.00%) |
|  | Intraocular pressure test abnormal | 5 ( 0.1%) | 3 ( 0.7%) | 1 ( 0.2%) | 0 ( 0.00%) | 1 ( 0.5%) |
|  | Glycosylated haemoglobin increased | 4 ( 0.1%) | 1 ( 0.2%) | 0 ( 0.00%) | 3 ( 0.6%) | 0 ( 0.00%) |
|  | Blood pressure abnormal | 2 ( 0.0%) | 0 ( 0.00%) | 1 ( 0.2%) | 1 ( 0.2%) | 0 ( 0.00%) |
|  | Blood pressure systolic increased | 2 ( 0.0%) | 0 ( 0.00%) | 0 ( 0.00%) | 0 ( 0.00%) | 2 ( 0.9%) |
|  | Catheterisation cardiac | 2 ( 0.0%) | 1 ( 0.2%) | 1 ( 0.2%) | 0 ( 0.00%) | 0 ( 0.00%) |
|  | International normalised ratio increased | 2 ( 0.0%) | 0 ( 0.00%) | 1 ( 0.2%) | 1 ( 0.2%) | 0 ( 0.00%) |
|  | Intraocular pressure fluctuation | 2 ( 0.0%) | 1 ( 0.2%) | 1 ( 0.2%) | 0 ( 0.00%) | 0 ( 0.00%) |
|  | Angiogram retina abnormal | 1 ( 0.0%) | 0 ( 0.00%) | 0 ( 0.00%) | 1 ( 0.2%) | 0 ( 0.00%) |
|  | Biopsy liver | 1 ( 0.0%) | 0 ( 0.00%) | 1 ( 0.2%) | 0 ( 0.00%) | 0 ( 0.00%) |
|  | Blood cholesterol increased | 1 ( 0.0%) | 1 ( 0.2%) | 0 ( 0.00%) | 0 ( 0.00%) | 0 ( 0.00%) |
|  | Blood creatine phosphokinase increased | 1 ( 0.0%) | 0 ( 0.00%) | 0 ( 0.00%) | 1 ( 0.2%) | 0 ( 0.00%) |
|  | Blood creatinine increased | 1 ( 0.0%) | 1 ( 0.2%) | 0 ( 0.00%) | 0 ( 0.00%) | 0 ( 0.00%) |
|  | Blood culture positive | 1 ( 0.0%) | 0 ( 0.00%) | 1 ( 0.2%) | 0 ( 0.00%) | 0 ( 0.00%) |
|  | Blood lactic acid increased | 1 ( 0.0%) | 0 ( 0.00%) | 0 ( 0.00%) | 1 ( 0.2%) | 0 ( 0.00%) |
|  | Blood pressure increased | 1 ( 0.0%) | 0 ( 0.00%) | 1 ( 0.2%) | 0 ( 0.00%) | 0 ( 0.00%) |
|  | Blood thyroid stimulating hormone decreased | 1 ( 0.0%) | 0 ( 0.00%) | 0 ( 0.00%) | 1 ( 0.2%) | 0 ( 0.00%) |
|  | C-reactive protein increased | 1 ( 0.0%) | 0 ( 0.00%) | 0 ( 0.00%) | 1 ( 0.2%) | 0 ( 0.00%) |
|  | Cystoscopy | 1 ( 0.0%) | 1 ( 0.2%) | 0 ( 0.00%) | 0 ( 0.00%) | 0 ( 0.00%) |
|  | Full blood count increased | 1 ( 0.0%) | 0 ( 0.00%) | 1 ( 0.2%) | 0 ( 0.00%) | 0 ( 0.00%) |
|  | General physical condition abnormal | 1 ( 0.0%) | 1 ( 0.2%) | 0 ( 0.00%) | 0 ( 0.00%) | 0 ( 0.00%) |
|  | Glycosylated haemoglobin | 1 ( 0.0%) | 0 ( 0.00%) | 0 ( 0.00%) | 0 ( 0.00%) | 1 ( 0.5%) |
|  | Haemoglobin decreased | 1 ( 0.0%) | 0 ( 0.00%) | 1 ( 0.2%) | 0 ( 0.00%) | 0 ( 0.00%) |
|  | Hepatic enzyme increased | 1 ( 0.0%) | 0 ( 0.00%) | 1 ( 0.2%) | 0 ( 0.00%) | 0 ( 0.00%) |
|  | Intraocular pressure test | 1 ( 0.0%) | 0 ( 0.00%) | 1 ( 0.2%) | 0 ( 0.00%) | 0 ( 0.00%) |
|  | Norovirus test positive | 1 ( 0.0%) | 0 ( 0.00%) | 1 ( 0.2%) | 0 ( 0.00%) | 0 ( 0.00%) |
|  | Visual acuity tests abnormal | 1 ( 0.0%) | 0 ( 0.00%) | 0 ( 0.00%) | 1 ( 0.2%) | 0 ( 0.00%) |
|  | Weight decreased | 1 ( 0.0%) | 0 ( 0.00%) | 1 ( 0.2%) | 0 ( 0.00%) | 0 ( 0.00%) |
|  | White blood cell count decreased | 1 ( 0.0%) | 1 ( 0.2%) | 0 ( 0.00%) | 0 ( 0.00%) | 0 ( 0.00%) |
|  |  |  |  |  |  |  |
| Surgical and medical procedures | All Patients | 153 ( 2.6%) | 90 ( 20.0%) | 29 ( 7.0%) | 22 ( 4.4%) | 15 ( 7.0%) |
|  | Cataract operation | 75 ( 1.3%) | 57 ( 12.6%) | 5 ( 1.2%) | 13 ( 2.6%) | 0 ( 0.00%) |
|  | Drug therapy | 14 ( 0.2%) | 9 ( 2.0%) | 2 ( 0.5%) | 4 ( 0.8%) | 0 ( 0.00%) |
|  | Hospitalisation | 12 ( 0.2%) | 1 ( 0.2%) | 3 ( 0.7%) | 0 ( 0.00%) | 9 ( 4.2%) |
|  | Laser therapy | 11 ( 0.2%) | 7 ( 1.6%) | 0 ( 0.00%) | 4 ( 0.8%) | 0 ( 0.00%) |
|  | Vitrectomy | 10 ( 0.2%) | 3 ( 0.7%) | 6 ( 1.4%) | 0 ( 0.00%) | 1 ( 0.5%) |
|  | Therapy change | 5 ( 0.1%) | 5 ( 1.1%) | 0 ( 0.00%) | 0 ( 0.00%) | 0 ( 0.00%) |
|  | Hip surgery | 3 ( 0.1%) | 1 ( 0.2%) | 2 ( 0.5%) | 0 ( 0.00%) | 0 ( 0.00%) |
|  | Intraocular lens implant | 3 ( 0.1%) | 2 ( 0.4%) | 1 ( 0.2%) | 0 ( 0.00%) | 0 ( 0.00%) |
|  | Knee operation | 3 ( 0.1%) | 2 ( 0.4%) | 0 ( 0.00%) | 0 ( 0.00%) | 1 ( 0.5%) |
|  | Prophylaxis | 3 ( 0.1%) | 2 ( 0.4%) | 0 ( 0.00%) | 1 ( 0.2%) | 0 ( 0.00%) |
|  | Cardiac pacemaker insertion | 2 ( 0.0%) | 1 ( 0.2%) | 1 ( 0.2%) | 0 ( 0.00%) | 0 ( 0.00%) |
|  | Heart valve operation | 2 ( 0.0%) | 0 ( 0.00%) | 2 ( 0.5%) | 0 ( 0.00%) | 0 ( 0.00%) |
|  | Lens extraction | 2 ( 0.0%) | 1 ( 0.2%) | 1 ( 0.2%) | 0 ( 0.00%) | 0 ( 0.00%) |
|  | Toe amputation | 2 ( 0.0%) | 0 ( 0.00%) | 2 ( 0.5%) | 0 ( 0.00%) | 0 ( 0.00%) |
|  | Vascular graft | 2 ( 0.0%) | 0 ( 0.00%) | 1 ( 0.2%) | 0 ( 0.00%) | 1 ( 0.5%) |
|  | Arterial repair | 1 ( 0.0%) | 0 ( 0.00%) | 1 ( 0.2%) | 0 ( 0.00%) | 0 ( 0.00%) |
|  | Blepharoplasty | 1 ( 0.0%) | 1 ( 0.2%) | 0 ( 0.00%) | 0 ( 0.00%) | 0 ( 0.00%) |
|  | Bunion operation | 1 ( 0.0%) | 1 ( 0.2%) | 0 ( 0.00%) | 0 ( 0.00%) | 0 ( 0.00%) |
|  | Cardiac operation | 1 ( 0.0%) | 0 ( 0.00%) | 0 ( 0.00%) | 0 ( 0.00%) | 1 ( 0.5%) |
|  | Cholecystectomy | 1 ( 0.0%) | 0 ( 0.00%) | 1 ( 0.2%) | 0 ( 0.00%) | 0 ( 0.00%) |
|  | Ciliary body operation | 1 ( 0.0%) | 0 ( 0.00%) | 1 ( 0.2%) | 0 ( 0.00%) | 0 ( 0.00%) |
|  | Coronary artery bypass | 1 ( 0.0%) | 1 ( 0.2%) | 0 ( 0.00%) | 0 ( 0.00%) | 0 ( 0.00%) |
|  | Dental implantation | 1 ( 0.0%) | 1 ( 0.2%) | 0 ( 0.00%) | 0 ( 0.00%) | 0 ( 0.00%) |
|  | Elective surgery | 1 ( 0.0%) | 1 ( 0.2%) | 0 ( 0.00%) | 0 ( 0.00%) | 0 ( 0.00%) |
|  | Eye operation | 1 ( 0.0%) | 0 ( 0.00%) | 1 ( 0.2%) | 0 ( 0.00%) | 0 ( 0.00%) |
|  | Hernia repair | 1 ( 0.0%) | 0 ( 0.00%) | 1 ( 0.2%) | 0 ( 0.00%) | 0 ( 0.00%) |
|  | Hysterectomy | 1 ( 0.0%) | 0 ( 0.00%) | 1 ( 0.2%) | 0 ( 0.00%) | 0 ( 0.00%) |
|  | Iridotomy | 1 ( 0.0%) | 0 ( 0.00%) | 0 ( 0.00%) | 1 ( 0.2%) | 0 ( 0.00%) |
|  | Knee arthroplasty | 1 ( 0.0%) | 0 ( 0.00%) | 1 ( 0.2%) | 0 ( 0.00%) | 0 ( 0.00%) |
|  | Ophthalmologic treatment | 1 ( 0.0%) | 0 ( 0.00%) | 1 ( 0.2%) | 0 ( 0.00%) | 0 ( 0.00%) |
|  | Oxygen supplementation | 1 ( 0.0%) | 0 ( 0.00%) | 0 ( 0.00%) | 0 ( 0.00%) | 1 ( 0.5%) |
|  | Radiotherapy | 1 ( 0.0%) | 0 ( 0.00%) | 1 ( 0.2%) | 0 ( 0.00%) | 0 ( 0.00%) |
|  | Retinal operation | 1 ( 0.0%) | 1 ( 0.2%) | 0 ( 0.00%) | 0 ( 0.00%) | 0 ( 0.00%) |
|  | Shoulder operation | 1 ( 0.0%) | 0 ( 0.00%) | 0 ( 0.00%) | 0 ( 0.00%) | 1 ( 0.5%) |
|  | Spinal cord operation | 1 ( 0.0%) | 0 ( 0.00%) | 1 ( 0.2%) | 0 ( 0.00%) | 0 ( 0.00%) |
|  | Spinal operation | 1 ( 0.0%) | 0 ( 0.00%) | 1 ( 0.2%) | 0 ( 0.00%) | 0 ( 0.00%) |
|  | Suture fixation of intraocular lens | 1 ( 0.0%) | 0 ( 0.00%) | 1 ( 0.2%) | 0 ( 0.00%) | 0 ( 0.00%) |
|  | Suture removal | 1 ( 0.0%) | 1 ( 0.2%) | 0 ( 0.00%) | 0 ( 0.00%) | 0 ( 0.00%) |
|  | Tendon operation | 1 ( 0.0%) | 1 ( 0.2%) | 0 ( 0.00%) | 0 ( 0.00%) | 0 ( 0.00%) |
|  | Therapeutic procedure | 1 ( 0.0%) | 0 ( 0.00%) | 1 ( 0.2%) | 0 ( 0.00%) | 0 ( 0.00%) |
|  | Tooth extraction | 1 ( 0.0%) | 1 ( 0.2%) | 0 ( 0.00%) | 0 ( 0.00%) | 0 ( 0.00%) |
|  |  |  |  |  |  |  |
| Infections and infestations | All Patients | 130 ( 2.2%) | 68 ( 15.1%) | 46 ( 11.0%) | 12 ( 2.4%) | 9 ( 4.2%) |
|  | Conjunctivitis | 29 ( 0.5%) | 25 ( 5.5%) | 0 ( 0.00%) | 4 ( 0.8%) | 0 ( 0.00%) |
|  | Pneumonia | 27 ( 0.5%) | 2 ( 0.4%) | 22 ( 5.3%) | 1 ( 0.2%) | 2 ( 0.9%) |
|  | Nasopharyngitis | 20 ( 0.3%) | 17 ( 3.8%) | 0 ( 0.00%) | 3 ( 0.6%) | 0 ( 0.00%) |
|  | Influenza | 9 ( 0.2%) | 7 ( 1.6%) | 1 ( 0.2%) | 1 ( 0.2%) | 0 ( 0.00%) |
|  | Endophthalmitis | 7 ( 0.1%) | 0 ( 0.00%) | 1 ( 0.2%) | 0 ( 0.00%) | 6 ( 2.8%) |
|  | Hordeolum | 6 ( 0.1%) | 5 ( 1.1%) | 1 ( 0.2%) | 0 ( 0.00%) | 0 ( 0.00%) |
|  | Sepsis | 5 ( 0.1%) | 0 ( 0.00%) | 5 ( 1.2%) | 0 ( 0.00%) | 0 ( 0.00%) |
|  | Ophthalmic herpes simplex | 4 ( 0.1%) | 1 ( 0.2%) | 1 ( 0.2%) | 1 ( 0.2%) | 1 ( 0.5%) |
|  | Respiratory tract infection | 4 ( 0.1%) | 1 ( 0.2%) | 3 ( 0.7%) | 0 ( 0.00%) | 0 ( 0.00%) |
|  | Bronchitis | 3 ( 0.1%) | 3 ( 0.7%) | 0 ( 0.00%) | 0 ( 0.00%) | 0 ( 0.00%) |
|  | Gastroenteritis | 3 ( 0.1%) | 3 ( 0.7%) | 0 ( 0.00%) | 0 ( 0.00%) | 0 ( 0.00%) |
|  | Herpes zoster | 3 ( 0.1%) | 3 ( 0.7%) | 0 ( 0.00%) | 0 ( 0.00%) | 0 ( 0.00%) |
|  | Infection | 3 ( 0.1%) | 2 ( 0.4%) | 1 ( 0.2%) | 0 ( 0.00%) | 0 ( 0.00%) |
|  | Febrile infection | 2 ( 0.0%) | 0 ( 0.00%) | 2 ( 0.5%) | 0 ( 0.00%) | 0 ( 0.00%) |
|  | Pyelitis | 2 ( 0.0%) | 0 ( 0.00%) | 2 ( 0.5%) | 0 ( 0.00%) | 0 ( 0.00%) |
|  | Abscess | 1 ( 0.0%) | 0 ( 0.00%) | 0 ( 0.00%) | 1 ( 0.2%) | 0 ( 0.00%) |
|  | Bacterial infection | 1 ( 0.0%) | 0 ( 0.00%) | 1 ( 0.2%) | 0 ( 0.00%) | 0 ( 0.00%) |
|  | Cellulitis | 1 ( 0.0%) | 0 ( 0.00%) | 1 ( 0.2%) | 0 ( 0.00%) | 0 ( 0.00%) |
|  | Conjunctivitis bacterial | 1 ( 0.0%) | 1 ( 0.2%) | 0 ( 0.00%) | 0 ( 0.00%) | 0 ( 0.00%) |
|  | Cystitis | 1 ( 0.0%) | 1 ( 0.2%) | 0 ( 0.00%) | 0 ( 0.00%) | 0 ( 0.00%) |
|  | Diabetic foot infection | 1 ( 0.0%) | 0 ( 0.00%) | 1 ( 0.2%) | 0 ( 0.00%) | 0 ( 0.00%) |
|  | Ear infection | 1 ( 0.0%) | 1 ( 0.2%) | 0 ( 0.00%) | 0 ( 0.00%) | 0 ( 0.00%) |
|  | Endocarditis | 1 ( 0.0%) | 0 ( 0.00%) | 0 ( 0.00%) | 0 ( 0.00%) | 1 ( 0.5%) |
|  | Epididymitis | 1 ( 0.0%) | 1 ( 0.2%) | 0 ( 0.00%) | 0 ( 0.00%) | 0 ( 0.00%) |
|  | Erysipelas | 1 ( 0.0%) | 0 ( 0.00%) | 1 ( 0.2%) | 0 ( 0.00%) | 0 ( 0.00%) |
|  | Gastrointestinal fungal infection | 1 ( 0.0%) | 1 ( 0.2%) | 0 ( 0.00%) | 0 ( 0.00%) | 0 ( 0.00%) |
|  | Gastrointestinal infection | 1 ( 0.0%) | 1 ( 0.2%) | 0 ( 0.00%) | 0 ( 0.00%) | 0 ( 0.00%) |
|  | Gingivitis | 1 ( 0.0%) | 1 ( 0.2%) | 0 ( 0.00%) | 0 ( 0.00%) | 0 ( 0.00%) |
|  | Helicobacter infection | 1 ( 0.0%) | 1 ( 0.2%) | 0 ( 0.00%) | 0 ( 0.00%) | 0 ( 0.00%) |
|  | Hypopyon | 1 ( 0.0%) | 0 ( 0.00%) | 1 ( 0.2%) | 0 ( 0.00%) | 0 ( 0.00%) |
|  | Lyme disease | 1 ( 0.0%) | 0 ( 0.00%) | 1 ( 0.2%) | 0 ( 0.00%) | 0 ( 0.00%) |
|  | Nipple infection | 1 ( 0.0%) | 1 ( 0.2%) | 0 ( 0.00%) | 0 ( 0.00%) | 0 ( 0.00%) |
|  | Oesophageal candidiasis | 1 ( 0.0%) | 0 ( 0.00%) | 0 ( 0.00%) | 1 ( 0.2%) | 0 ( 0.00%) |
|  | Oral herpes | 1 ( 0.0%) | 0 ( 0.00%) | 0 ( 0.00%) | 1 ( 0.2%) | 0 ( 0.00%) |
|  | Perirectal abscess | 1 ( 0.0%) | 0 ( 0.00%) | 1 ( 0.2%) | 0 ( 0.00%) | 0 ( 0.00%) |
|  | Pulpitis dental | 1 ( 0.0%) | 1 ( 0.2%) | 0 ( 0.00%) | 0 ( 0.00%) | 0 ( 0.00%) |
|  | Purulence | 1 ( 0.0%) | 1 ( 0.2%) | 0 ( 0.00%) | 0 ( 0.00%) | 0 ( 0.00%) |
|  | Rotavirus infection | 1 ( 0.0%) | 0 ( 0.00%) | 1 ( 0.2%) | 0 ( 0.00%) | 0 ( 0.00%) |
|  | Septic shock | 1 ( 0.0%) | 0 ( 0.00%) | 0 ( 0.00%) | 0 ( 0.00%) | 1 ( 0.5%) |
|  | Sinusitis | 1 ( 0.0%) | 1 ( 0.2%) | 0 ( 0.00%) | 0 ( 0.00%) | 0 ( 0.00%) |
|  | Tonsillitis | 1 ( 0.0%) | 1 ( 0.2%) | 0 ( 0.00%) | 0 ( 0.00%) | 0 ( 0.00%) |
|  | Urosepsis | 1 ( 0.0%) | 0 ( 0.00%) | 1 ( 0.2%) | 0 ( 0.00%) | 0 ( 0.00%) |
|  | Wound infection | 1 ( 0.0%) | 0 ( 0.00%) | 0 ( 0.00%) | 1 ( 0.2%) | 0 ( 0.00%) |
|  | Wound infection bacterial | 1 ( 0.0%) | 0 ( 0.00%) | 1 ( 0.2%) | 0 ( 0.00%) | 0 ( 0.00%) |
|  | Wound sepsis | 1 ( 0.0%) | 0 ( 0.00%) | 1 ( 0.2%) | 0 ( 0.00%) | 0 ( 0.00%) |
|  |  |  |  |  |  |  |
| Injury, poisoning and procedural complications | All Patients | 106 ( 1.8%) | 23 ( 5.1%) | 50 ( 12.0%) | 29 ( 5.8%) | 12 ( 5.6%) |
|  | Fall | 43 ( 0.7%) | 11 ( 2.4%) | 24 ( 5.8%) | 2 ( 0.4%) | 6 ( 2.8%) |
|  | Inappropriate schedule of drug administration | 18 ( 0.3%) | 2 ( 0.4%) | 0 ( 0.00%) | 16 ( 3.2%) | 0 ( 0.00%) |
|  | Femoral neck fracture | 14 ( 0.2%) | 0 ( 0.00%) | 13 ( 3.1%) | 0 ( 0.00%) | 1 ( 0.5%) |
|  | Spinal fracture | 4 ( 0.1%) | 0 ( 0.00%) | 4 ( 1.0%) | 0 ( 0.00%) | 0 ( 0.00%) |
|  | Upper limb fracture | 4 ( 0.1%) | 3 ( 0.7%) | 1 ( 0.2%) | 0 ( 0.00%) | 1 ( 0.5%) |
|  | Drug administered at inappropriate site | 3 ( 0.1%) | 0 ( 0.00%) | 0 ( 0.00%) | 3 ( 0.6%) | 0 ( 0.00%) |
|  | Ligament sprain | 3 ( 0.1%) | 3 ( 0.7%) | 0 ( 0.00%) | 0 ( 0.00%) | 0 ( 0.00%) |
|  | Multiple use of single-use product | 3 ( 0.1%) | 0 ( 0.00%) | 0 ( 0.00%) | 3 ( 0.6%) | 0 ( 0.00%) |
|  | Accident | 2 ( 0.0%) | 0 ( 0.00%) | 2 ( 0.5%) | 0 ( 0.00%) | 0 ( 0.00%) |
|  | Craniocerebral injury | 2 ( 0.0%) | 0 ( 0.00%) | 2 ( 0.5%) | 0 ( 0.00%) | 0 ( 0.00%) |
|  | Eyelid injury | 2 ( 0.0%) | 2 ( 0.4%) | 0 ( 0.00%) | 0 ( 0.00%) | 0 ( 0.00%) |
|  | Fracture | 2 ( 0.0%) | 0 ( 0.00%) | 1 ( 0.2%) | 0 ( 0.00%) | 1 ( 0.5%) |
|  | Lower limb fracture | 2 ( 0.0%) | 0 ( 0.00%) | 2 ( 0.5%) | 0 ( 0.00%) | 0 ( 0.00%) |
|  | Lumbar vertebral fracture | 2 ( 0.0%) | 0 ( 0.00%) | 2 ( 0.5%) | 0 ( 0.00%) | 0 ( 0.00%) |
|  | Overdose | 2 ( 0.0%) | 0 ( 0.00%) | 0 ( 0.00%) | 2 ( 0.4%) | 0 ( 0.00%) |
|  | Rib fracture | 2 ( 0.0%) | 0 ( 0.00%) | 2 ( 0.5%) | 0 ( 0.00%) | 0 ( 0.00%) |
|  | Wound | 2 ( 0.0%) | 0 ( 0.00%) | 1 ( 0.2%) | 1 ( 0.2%) | 0 ( 0.00%) |
|  | Arthropod bite | 1 ( 0.0%) | 1 ( 0.2%) | 0 ( 0.00%) | 0 ( 0.00%) | 0 ( 0.00%) |
|  | Cardiac valve rupture | 1 ( 0.0%) | 0 ( 0.00%) | 0 ( 0.00%) | 0 ( 0.00%) | 1 ( 0.5%) |
|  | Contusion | 1 ( 0.0%) | 1 ( 0.2%) | 0 ( 0.00%) | 0 ( 0.00%) | 0 ( 0.00%) |
|  | Drug administration error | 1 ( 0.0%) | 0 ( 0.00%) | 0 ( 0.00%) | 1 ( 0.2%) | 0 ( 0.00%) |
|  | Eye burns | 1 ( 0.0%) | 1 ( 0.2%) | 0 ( 0.00%) | 0 ( 0.00%) | 0 ( 0.00%) |
|  | Eye contusion | 1 ( 0.0%) | 0 ( 0.00%) | 0 ( 0.00%) | 0 ( 0.00%) | 1 ( 0.5%) |
|  | Eye injury | 1 ( 0.0%) | 1 ( 0.2%) | 0 ( 0.00%) | 0 ( 0.00%) | 0 ( 0.00%) |
|  | Eye laser scar | 1 ( 0.0%) | 1 ( 0.2%) | 0 ( 0.00%) | 0 ( 0.00%) | 0 ( 0.00%) |
|  | Facial bones fracture | 1 ( 0.0%) | 0 ( 0.00%) | 1 ( 0.2%) | 0 ( 0.00%) | 0 ( 0.00%) |
|  | Femur fracture | 1 ( 0.0%) | 0 ( 0.00%) | 1 ( 0.2%) | 0 ( 0.00%) | 0 ( 0.00%) |
|  | Foot fracture | 1 ( 0.0%) | 0 ( 0.00%) | 1 ( 0.2%) | 0 ( 0.00%) | 0 ( 0.00%) |
|  | Injury corneal | 1 ( 0.0%) | 1 ( 0.2%) | 0 ( 0.00%) | 0 ( 0.00%) | 0 ( 0.00%) |
|  | Open globe injury | 1 ( 0.0%) | 0 ( 0.00%) | 1 ( 0.2%) | 0 ( 0.00%) | 0 ( 0.00%) |
|  | Pelvic fracture | 1 ( 0.0%) | 0 ( 0.00%) | 1 ( 0.2%) | 0 ( 0.00%) | 0 ( 0.00%) |
|  | Periorbital haematoma | 1 ( 0.0%) | 0 ( 0.00%) | 1 ( 0.2%) | 0 ( 0.00%) | 0 ( 0.00%) |
|  | Post procedural haematoma | 1 ( 0.0%) | 0 ( 0.00%) | 1 ( 0.2%) | 0 ( 0.00%) | 0 ( 0.00%) |
|  | Prescribed overdose | 1 ( 0.0%) | 0 ( 0.00%) | 0 ( 0.00%) | 1 ( 0.2%) | 0 ( 0.00%) |
|  | Procedural pain | 1 ( 0.0%) | 1 ( 0.2%) | 0 ( 0.00%) | 0 ( 0.00%) | 0 ( 0.00%) |
|  | Retinal injury | 1 ( 0.0%) | 0 ( 0.00%) | 0 ( 0.00%) | 0 ( 0.00%) | 1 ( 0.5%) |
|  | Scar | 1 ( 0.0%) | 0 ( 0.00%) | 1 ( 0.2%) | 0 ( 0.00%) | 0 ( 0.00%) |
|  | Skin injury | 1 ( 0.0%) | 0 ( 0.00%) | 1 ( 0.2%) | 0 ( 0.00%) | 0 ( 0.00%) |
|  | Spinal column injury | 1 ( 0.0%) | 0 ( 0.00%) | 1 ( 0.2%) | 0 ( 0.00%) | 0 ( 0.00%) |
|  | Spinal cord injury cervical | 1 ( 0.0%) | 0 ( 0.00%) | 1 ( 0.2%) | 0 ( 0.00%) | 0 ( 0.00%) |
|  | Subarachnoid haemorrhage | 1 ( 0.0%) | 0 ( 0.00%) | 1 ( 0.2%) | 0 ( 0.00%) | 0 ( 0.00%) |
|  | Subdural haematoma | 1 ( 0.0%) | 0 ( 0.00%) | 1 ( 0.2%) | 0 ( 0.00%) | 0 ( 0.00%) |
|  | Thoracic vertebral fracture | 1 ( 0.0%) | 0 ( 0.00%) | 1 ( 0.2%) | 0 ( 0.00%) | 0 ( 0.00%) |
|  | Toxic anterior segment syndrome | 1 ( 0.0%) | 0 ( 0.00%) | 0 ( 0.00%) | 0 ( 0.00%) | 1 ( 0.5%) |
|  | Traumatic haematoma | 1 ( 0.0%) | 1 ( 0.2%) | 0 ( 0.00%) | 0 ( 0.00%) | 0 ( 0.00%) |
|  |  |  |  |  |  |  |
| Nervous system disorders | All Patients | 85 ( 1.5%) | 12 ( 2.7%) | 38 ( 9.1%) | 7 ( 1.4%) | 30 ( 14.1%) |
|  | Cerebrovascular accident | 27 ( 0.5%) | 0 ( 0.00%) | 11 ( 2.6%) | 0 ( 0.00%) | 16 ( 7.5%) |
|  | Dizziness | 10 ( 0.2%) | 2 ( 0.4%) | 4 ( 1.0%) | 1 ( 0.2%) | 3 ( 1.4%) |
|  | Motor dysfunction | 8 ( 0.1%) | 0 ( 0.00%) | 3 ( 0.7%) | 0 ( 0.00%) | 5 ( 2.3%) |
|  | Aphasia | 5 ( 0.1%) | 0 ( 0.00%) | 2 ( 0.5%) | 0 ( 0.00%) | 3 ( 1.4%) |
|  | Coordination abnormal | 5 ( 0.1%) | 0 ( 0.00%) | 4 ( 1.0%) | 0 ( 0.00%) | 1 ( 0.5%) |
|  | Dementia | 4 ( 0.1%) | 0 ( 0.00%) | 3 ( 0.7%) | 0 ( 0.00%) | 1 ( 0.5%) |
|  | Headache | 4 ( 0.1%) | 2 ( 0.4%) | 0 ( 0.00%) | 0 ( 0.00%) | 2 ( 0.9%) |
|  | Syncope | 4 ( 0.1%) | 1 ( 0.2%) | 3 ( 0.7%) | 0 ( 0.00%) | 0 ( 0.00%) |
|  | Visual field defect | 4 ( 0.1%) | 0 ( 0.00%) | 1 ( 0.2%) | 2 ( 0.4%) | 1 ( 0.5%) |
|  | Loss of consciousness | 3 ( 0.1%) | 0 ( 0.00%) | 1 ( 0.2%) | 0 ( 0.00%) | 2 ( 0.9%) |
|  | Paralysis | 3 ( 0.1%) | 0 ( 0.00%) | 3 ( 0.7%) | 0 ( 0.00%) | 0 ( 0.00%) |
|  | Balance disorder | 2 ( 0.0%) | 0 ( 0.00%) | 1 ( 0.2%) | 0 ( 0.00%) | 1 ( 0.5%) |
|  | Carotid artery stenosis | 2 ( 0.0%) | 0 ( 0.00%) | 2 ( 0.5%) | 0 ( 0.00%) | 0 ( 0.00%) |
|  | Cerebral infarction | 2 ( 0.0%) | 0 ( 0.00%) | 0 ( 0.00%) | 0 ( 0.00%) | 2 ( 0.9%) |
|  | Diabetic neuropathy | 2 ( 0.0%) | 0 ( 0.00%) | 2 ( 0.5%) | 0 ( 0.00%) | 0 ( 0.00%) |
|  | Hypoaesthesia | 2 ( 0.0%) | 0 ( 0.00%) | 1 ( 0.2%) | 1 ( 0.2%) | 0 ( 0.00%) |
|  | Ischaemic stroke | 2 ( 0.0%) | 0 ( 0.00%) | 0 ( 0.00%) | 0 ( 0.00%) | 2 ( 0.9%) |
|  | Parkinson's disease | 2 ( 0.0%) | 0 ( 0.00%) | 2 ( 0.5%) | 0 ( 0.00%) | 0 ( 0.00%) |
|  | Senile dementia | 2 ( 0.0%) | 0 ( 0.00%) | 1 ( 0.2%) | 1 ( 0.2%) | 0 ( 0.00%) |
|  | Spinal cord haemorrhage | 2 ( 0.0%) | 0 ( 0.00%) | 1 ( 0.2%) | 0 ( 0.00%) | 1 ( 0.5%) |
|  | Transient ischaemic attack | 2 ( 0.0%) | 0 ( 0.00%) | 0 ( 0.00%) | 0 ( 0.00%) | 2 ( 0.9%) |
|  | Tremor | 2 ( 0.0%) | 1 ( 0.2%) | 0 ( 0.00%) | 1 ( 0.2%) | 0 ( 0.00%) |
|  | Carotid artery thrombosis | 1 ( 0.0%) | 0 ( 0.00%) | 1 ( 0.2%) | 0 ( 0.00%) | 0 ( 0.00%) |
|  | Cerebral haemorrhage | 1 ( 0.0%) | 0 ( 0.00%) | 1 ( 0.2%) | 0 ( 0.00%) | 0 ( 0.00%) |
|  | Cerebral hypoperfusion | 1 ( 0.0%) | 0 ( 0.00%) | 1 ( 0.2%) | 0 ( 0.00%) | 0 ( 0.00%) |
|  | Cerebral ischaemia | 1 ( 0.0%) | 0 ( 0.00%) | 0 ( 0.00%) | 0 ( 0.00%) | 1 ( 0.5%) |
|  | Dementia Alzheimer's type | 1 ( 0.0%) | 0 ( 0.00%) | 1 ( 0.2%) | 0 ( 0.00%) | 0 ( 0.00%) |
|  | Embolic stroke | 1 ( 0.0%) | 0 ( 0.00%) | 1 ( 0.2%) | 0 ( 0.00%) | 0 ( 0.00%) |
|  | Epilepsy | 1 ( 0.0%) | 0 ( 0.00%) | 1 ( 0.2%) | 0 ( 0.00%) | 0 ( 0.00%) |
|  | Facial paralysis | 1 ( 0.0%) | 1 ( 0.2%) | 0 ( 0.00%) | 0 ( 0.00%) | 0 ( 0.00%) |
|  | Gliosis | 1 ( 0.0%) | 0 ( 0.00%) | 0 ( 0.00%) | 1 ( 0.2%) | 0 ( 0.00%) |
|  | Hemiparesis | 1 ( 0.0%) | 0 ( 0.00%) | 0 ( 0.00%) | 0 ( 0.00%) | 1 ( 0.5%) |
|  | Hemiplegia | 1 ( 0.0%) | 0 ( 0.00%) | 1 ( 0.2%) | 0 ( 0.00%) | 0 ( 0.00%) |
|  | Hydrocephalus | 1 ( 0.0%) | 0 ( 0.00%) | 1 ( 0.2%) | 0 ( 0.00%) | 0 ( 0.00%) |
|  | IIIrd nerve paralysis | 1 ( 0.0%) | 0 ( 0.00%) | 1 ( 0.2%) | 0 ( 0.00%) | 0 ( 0.00%) |
|  | IIIrd nerve paresis | 1 ( 0.0%) | 0 ( 0.00%) | 0 ( 0.00%) | 0 ( 0.00%) | 1 ( 0.5%) |
|  | Intraventricular haemorrhage | 1 ( 0.0%) | 0 ( 0.00%) | 1 ( 0.2%) | 0 ( 0.00%) | 0 ( 0.00%) |
|  | Migraine | 1 ( 0.0%) | 1 ( 0.2%) | 0 ( 0.00%) | 0 ( 0.00%) | 0 ( 0.00%) |
|  | Mononeuritis | 1 ( 0.0%) | 0 ( 0.00%) | 0 ( 0.00%) | 0 ( 0.00%) | 1 ( 0.5%) |
|  | Myasthenia gravis | 1 ( 0.0%) | 0 ( 0.00%) | 1 ( 0.2%) | 0 ( 0.00%) | 0 ( 0.00%) |
|  | Neuralgia | 1 ( 0.0%) | 1 ( 0.2%) | 0 ( 0.00%) | 0 ( 0.00%) | 0 ( 0.00%) |
|  | Normal pressure hydrocephalus | 1 ( 0.0%) | 0 ( 0.00%) | 1 ( 0.2%) | 0 ( 0.00%) | 0 ( 0.00%) |
|  | Paraesthesia | 1 ( 0.0%) | 1 ( 0.2%) | 0 ( 0.00%) | 0 ( 0.00%) | 0 ( 0.00%) |
|  | Partial seizures | 1 ( 0.0%) | 0 ( 0.00%) | 1 ( 0.2%) | 0 ( 0.00%) | 0 ( 0.00%) |
|  | Phrenic nerve paralysis | 1 ( 0.0%) | 0 ( 0.00%) | 0 ( 0.00%) | 0 ( 0.00%) | 1 ( 0.5%) |
|  | Sciatica | 1 ( 0.0%) | 1 ( 0.2%) | 0 ( 0.00%) | 0 ( 0.00%) | 0 ( 0.00%) |
|  | Sensorimotor disorder | 1 ( 0.0%) | 0 ( 0.00%) | 1 ( 0.2%) | 0 ( 0.00%) | 0 ( 0.00%) |
|  | Sensory loss | 1 ( 0.0%) | 0 ( 0.00%) | 0 ( 0.00%) | 0 ( 0.00%) | 1 ( 0.5%) |
|  | VIth nerve paralysis | 1 ( 0.0%) | 1 ( 0.2%) | 0 ( 0.00%) | 0 ( 0.00%) | 0 ( 0.00%) |
|  |  |  |  |  |  |  |
| Cardiac disorders | All Patients | 80 ( 1.4%) | 4 ( 0.9%) | 56 ( 13.4%) | 4 ( 0.8%) | 22 ( 10.3%) |
|  | Cardiac failure | 23 ( 0.4%) | 0 ( 0.00%) | 21 ( 5.0%) | 0 ( 0.00%) | 2 ( 0.9%) |
|  | Myocardial infarction | 16 ( 0.3%) | 0 ( 0.00%) | 11 ( 2.6%) | 0 ( 0.00%) | 5 ( 2.3%) |
|  | Angina pectoris | 7 ( 0.1%) | 0 ( 0.00%) | 1 ( 0.2%) | 0 ( 0.00%) | 6 ( 2.8%) |
|  | Atrial fibrillation | 7 ( 0.1%) | 0 ( 0.00%) | 4 ( 1.0%) | 0 ( 0.00%) | 3 ( 1.4%) |
|  | Cardiac arrest | 6 ( 0.1%) | 0 ( 0.00%) | 6 ( 1.4%) | 0 ( 0.00%) | 0 ( 0.00%) |
|  | Cardiac disorder | 6 ( 0.1%) | 0 ( 0.00%) | 6 ( 1.4%) | 0 ( 0.00%) | 0 ( 0.00%) |
|  | Coronary artery disease | 6 ( 0.1%) | 0 ( 0.00%) | 3 ( 0.7%) | 0 ( 0.00%) | 3 ( 1.4%) |
|  | Arrhythmia | 5 ( 0.1%) | 0 ( 0.00%) | 2 ( 0.5%) | 0 ( 0.00%) | 3 ( 1.4%) |
|  | Cardiovascular disorder | 5 ( 0.1%) | 3 ( 0.7%) | 2 ( 0.5%) | 0 ( 0.00%) | 0 ( 0.00%) |
|  | Acute myocardial infarction | 2 ( 0.0%) | 0 ( 0.00%) | 1 ( 0.2%) | 0 ( 0.00%) | 1 ( 0.5%) |
|  | Aortic valve stenosis | 2 ( 0.0%) | 0 ( 0.00%) | 1 ( 0.2%) | 0 ( 0.00%) | 1 ( 0.5%) |
|  | Supraventricular extrasystoles | 2 ( 0.0%) | 0 ( 0.00%) | 1 ( 0.2%) | 1 ( 0.2%) | 0 ( 0.00%) |
|  | Angina unstable | 1 ( 0.0%) | 0 ( 0.00%) | 1 ( 0.2%) | 0 ( 0.00%) | 0 ( 0.00%) |
|  | Atrial flutter | 1 ( 0.0%) | 0 ( 0.00%) | 1 ( 0.2%) | 0 ( 0.00%) | 0 ( 0.00%) |
|  | Atrial tachycardia | 1 ( 0.0%) | 0 ( 0.00%) | 1 ( 0.2%) | 0 ( 0.00%) | 0 ( 0.00%) |
|  | Atrioventricular block complete | 1 ( 0.0%) | 0 ( 0.00%) | 1 ( 0.2%) | 0 ( 0.00%) | 0 ( 0.00%) |
|  | Bradyarrhythmia | 1 ( 0.0%) | 0 ( 0.00%) | 1 ( 0.2%) | 0 ( 0.00%) | 0 ( 0.00%) |
|  | Cardiac failure acute | 1 ( 0.0%) | 0 ( 0.00%) | 1 ( 0.2%) | 0 ( 0.00%) | 0 ( 0.00%) |
|  | Cardiogenic shock | 1 ( 0.0%) | 0 ( 0.00%) | 1 ( 0.2%) | 0 ( 0.00%) | 0 ( 0.00%) |
|  | Cardiopulmonary failure | 1 ( 0.0%) | 0 ( 0.00%) | 1 ( 0.2%) | 0 ( 0.00%) | 0 ( 0.00%) |
|  | Cardiorenal syndrome | 1 ( 0.0%) | 0 ( 0.00%) | 1 ( 0.2%) | 0 ( 0.00%) | 0 ( 0.00%) |
|  | Cardiovascular insufficiency | 1 ( 0.0%) | 1 ( 0.2%) | 0 ( 0.00%) | 0 ( 0.00%) | 0 ( 0.00%) |
|  | Coronary artery insufficiency | 1 ( 0.0%) | 0 ( 0.00%) | 1 ( 0.2%) | 0 ( 0.00%) | 0 ( 0.00%) |
|  | Heart valve stenosis | 1 ( 0.0%) | 0 ( 0.00%) | 0 ( 0.00%) | 0 ( 0.00%) | 1 ( 0.5%) |
|  | Left ventricular hypertrophy | 1 ( 0.0%) | 0 ( 0.00%) | 0 ( 0.00%) | 1 ( 0.2%) | 0 ( 0.00%) |
|  | Mitral valve incompetence | 1 ( 0.0%) | 0 ( 0.00%) | 0 ( 0.00%) | 1 ( 0.2%) | 0 ( 0.00%) |
|  | Myocardial ischaemia | 1 ( 0.0%) | 0 ( 0.00%) | 1 ( 0.2%) | 0 ( 0.00%) | 0 ( 0.00%) |
|  | Myocarditis | 1 ( 0.0%) | 0 ( 0.00%) | 0 ( 0.00%) | 0 ( 0.00%) | 1 ( 0.5%) |
|  | Palpitations | 1 ( 0.0%) | 0 ( 0.00%) | 0 ( 0.00%) | 1 ( 0.2%) | 0 ( 0.00%) |
|  | Sinoatrial block | 1 ( 0.0%) | 0 ( 0.00%) | 1 ( 0.2%) | 0 ( 0.00%) | 0 ( 0.00%) |
|  | Tachycardia | 1 ( 0.0%) | 0 ( 0.00%) | 0 ( 0.00%) | 1 ( 0.2%) | 0 ( 0.00%) |
|  | Tricuspid valve disease | 1 ( 0.0%) | 0 ( 0.00%) | 0 ( 0.00%) | 1 ( 0.2%) | 0 ( 0.00%) |
|  |  |  |  |  |  |  |
| Neoplasms benign, malignant and unspecified (incl cysts and polyps) | All Patients | 51 ( 0.9%) | 0 ( 0.00%) | 44 ( 10.6%) | 1 ( 0.2%) | 7 ( 3.3%) |
|  | Gastrointestinal carcinoma | 8 ( 0.1%) | 0 ( 0.00%) | 6 ( 1.4%) | 0 ( 0.00%) | 2 ( 0.9%) |
|  | Bronchial carcinoma | 3 ( 0.1%) | 0 ( 0.00%) | 2 ( 0.5%) | 0 ( 0.00%) | 1 ( 0.5%) |
|  | Lung neoplasm malignant | 3 ( 0.1%) | 0 ( 0.00%) | 2 ( 0.5%) | 0 ( 0.00%) | 1 ( 0.5%) |
|  | Metastases to liver | 3 ( 0.1%) | 0 ( 0.00%) | 3 ( 0.7%) | 0 ( 0.00%) | 0 ( 0.00%) |
|  | Neoplasm malignant | 3 ( 0.1%) | 0 ( 0.00%) | 2 ( 0.5%) | 0 ( 0.00%) | 1 ( 0.5%) |
|  | Basal cell carcinoma | 2 ( 0.0%) | 0 ( 0.00%) | 1 ( 0.2%) | 1 ( 0.2%) | 0 ( 0.00%) |
|  | Bladder cancer | 2 ( 0.0%) | 0 ( 0.00%) | 1 ( 0.2%) | 0 ( 0.00%) | 1 ( 0.5%) |
|  | Breast cancer | 2 ( 0.0%) | 0 ( 0.00%) | 2 ( 0.5%) | 0 ( 0.00%) | 0 ( 0.00%) |
|  | Colon cancer | 2 ( 0.0%) | 0 ( 0.00%) | 2 ( 0.5%) | 0 ( 0.00%) | 0 ( 0.00%) |
|  | Eyelid tumour | 2 ( 0.0%) | 0 ( 0.00%) | 2 ( 0.5%) | 0 ( 0.00%) | 0 ( 0.00%) |
|  | Hepatocellular carcinoma | 2 ( 0.0%) | 0 ( 0.00%) | 2 ( 0.5%) | 0 ( 0.00%) | 0 ( 0.00%) |
|  | Prostate cancer | 2 ( 0.0%) | 0 ( 0.00%) | 1 ( 0.2%) | 0 ( 0.00%) | 1 ( 0.5%) |
|  | Adenocarcinoma | 1 ( 0.0%) | 0 ( 0.00%) | 1 ( 0.2%) | 0 ( 0.00%) | 0 ( 0.00%) |
|  | Bile duct cancer | 1 ( 0.0%) | 0 ( 0.00%) | 1 ( 0.2%) | 0 ( 0.00%) | 0 ( 0.00%) |
|  | Brain neoplasm | 1 ( 0.0%) | 0 ( 0.00%) | 1 ( 0.2%) | 0 ( 0.00%) | 0 ( 0.00%) |
|  | Breast cancer metastatic | 1 ( 0.0%) | 0 ( 0.00%) | 1 ( 0.2%) | 0 ( 0.00%) | 0 ( 0.00%) |
|  | Cervix carcinoma | 1 ( 0.0%) | 0 ( 0.00%) | 1 ( 0.2%) | 0 ( 0.00%) | 0 ( 0.00%) |
|  | Cholangiocarcinoma | 1 ( 0.0%) | 0 ( 0.00%) | 1 ( 0.2%) | 0 ( 0.00%) | 0 ( 0.00%) |
|  | Chronic lymphocytic leukaemia | 1 ( 0.0%) | 0 ( 0.00%) | 1 ( 0.2%) | 0 ( 0.00%) | 0 ( 0.00%) |
|  | Colon cancer metastatic | 1 ( 0.0%) | 0 ( 0.00%) | 1 ( 0.2%) | 0 ( 0.00%) | 0 ( 0.00%) |
|  | Gastric cancer | 1 ( 0.0%) | 0 ( 0.00%) | 0 ( 0.00%) | 0 ( 0.00%) | 1 ( 0.5%) |
|  | Hepatic cancer | 1 ( 0.0%) | 0 ( 0.00%) | 1 ( 0.2%) | 0 ( 0.00%) | 0 ( 0.00%) |
|  | Hepatic neoplasm | 1 ( 0.0%) | 0 ( 0.00%) | 1 ( 0.2%) | 0 ( 0.00%) | 0 ( 0.00%) |
|  | Laryngeal cancer | 1 ( 0.0%) | 0 ( 0.00%) | 1 ( 0.2%) | 0 ( 0.00%) | 0 ( 0.00%) |
|  | Malignant neoplasm progression | 1 ( 0.0%) | 0 ( 0.00%) | 1 ( 0.2%) | 0 ( 0.00%) | 0 ( 0.00%) |
|  | Meningioma | 1 ( 0.0%) | 0 ( 0.00%) | 1 ( 0.2%) | 0 ( 0.00%) | 0 ( 0.00%) |
|  | Metastases to bone | 1 ( 0.0%) | 0 ( 0.00%) | 1 ( 0.2%) | 0 ( 0.00%) | 0 ( 0.00%) |
|  | Metastases to lung | 1 ( 0.0%) | 0 ( 0.00%) | 1 ( 0.2%) | 0 ( 0.00%) | 0 ( 0.00%) |
|  | Metastases to lymph nodes | 1 ( 0.0%) | 0 ( 0.00%) | 1 ( 0.2%) | 0 ( 0.00%) | 0 ( 0.00%) |
|  | Metastasis | 1 ( 0.0%) | 0 ( 0.00%) | 1 ( 0.2%) | 0 ( 0.00%) | 0 ( 0.00%) |
|  | Metastatic bronchial carcinoma | 1 ( 0.0%) | 0 ( 0.00%) | 1 ( 0.2%) | 0 ( 0.00%) | 0 ( 0.00%) |
|  | Non-Hodgkin's lymphoma | 1 ( 0.0%) | 0 ( 0.00%) | 1 ( 0.2%) | 0 ( 0.00%) | 0 ( 0.00%) |
|  | Oesophageal carcinoma | 1 ( 0.0%) | 0 ( 0.00%) | 1 ( 0.2%) | 0 ( 0.00%) | 0 ( 0.00%) |
|  | Oligodendroglioma | 1 ( 0.0%) | 0 ( 0.00%) | 1 ( 0.2%) | 0 ( 0.00%) | 0 ( 0.00%) |
|  | Pancreatic carcinoma | 1 ( 0.0%) | 0 ( 0.00%) | 1 ( 0.2%) | 0 ( 0.00%) | 0 ( 0.00%) |
|  | Plasma cell myeloma | 1 ( 0.0%) | 0 ( 0.00%) | 1 ( 0.2%) | 0 ( 0.00%) | 0 ( 0.00%) |
|  | Renal cancer | 1 ( 0.0%) | 0 ( 0.00%) | 1 ( 0.2%) | 0 ( 0.00%) | 0 ( 0.00%) |
|  | Squamous cell carcinoma | 1 ( 0.0%) | 0 ( 0.00%) | 1 ( 0.2%) | 0 ( 0.00%) | 0 ( 0.00%) |
|  | Uterine cancer | 1 ( 0.0%) | 0 ( 0.00%) | 1 ( 0.2%) | 0 ( 0.00%) | 0 ( 0.00%) |
|  | Vulval cancer | 1 ( 0.0%) | 0 ( 0.00%) | 1 ( 0.2%) | 0 ( 0.00%) | 0 ( 0.00%) |
|  |  |  |  |  |  |  |
| Vascular disorders | All Patients | 45 ( 0.8%) | 11 ( 2.4%) | 19 ( 4.6%) | 6 ( 1.2%) | 10 ( 4.7%) |
|  | Hypertensive crisis | 9 ( 0.2%) | 0 ( 0.00%) | 5 ( 1.2%) | 0 ( 0.00%) | 4 ( 1.9%) |
|  | Hypertension | 7 ( 0.1%) | 3 ( 0.7%) | 0 ( 0.00%) | 3 ( 0.6%) | 1 ( 0.5%) |
|  | Neovascularisation | 3 ( 0.1%) | 2 ( 0.4%) | 1 ( 0.2%) | 0 ( 0.00%) | 0 ( 0.00%) |
|  | Thrombosis | 3 ( 0.1%) | 0 ( 0.00%) | 0 ( 0.00%) | 1 ( 0.2%) | 2 ( 0.9%) |
|  | Aortic aneurysm | 2 ( 0.0%) | 0 ( 0.00%) | 0 ( 0.00%) | 1 ( 0.2%) | 1 ( 0.5%) |
|  | Blood pressure fluctuation | 2 ( 0.0%) | 2 ( 0.4%) | 0 ( 0.00%) | 0 ( 0.00%) | 0 ( 0.00%) |
|  | Circulatory collapse | 2 ( 0.0%) | 0 ( 0.00%) | 2 ( 0.5%) | 0 ( 0.00%) | 0 ( 0.00%) |
|  | Deep vein thrombosis | 2 ( 0.0%) | 1 ( 0.2%) | 1 ( 0.2%) | 0 ( 0.00%) | 0 ( 0.00%) |
|  | Pallor | 2 ( 0.0%) | 0 ( 0.00%) | 1 ( 0.2%) | 0 ( 0.00%) | 1 ( 0.5%) |
|  | Peripheral arterial occlusive disease | 2 ( 0.0%) | 1 ( 0.2%) | 1 ( 0.2%) | 0 ( 0.00%) | 0 ( 0.00%) |
|  | Aneurysm | 1 ( 0.0%) | 0 ( 0.00%) | 1 ( 0.2%) | 0 ( 0.00%) | 0 ( 0.00%) |
|  | Arteriosclerosis | 1 ( 0.0%) | 1 ( 0.2%) | 0 ( 0.00%) | 0 ( 0.00%) | 0 ( 0.00%) |
|  | Blood pressure inadequately controlled | 1 ( 0.0%) | 0 ( 0.00%) | 1 ( 0.2%) | 0 ( 0.00%) | 0 ( 0.00%) |
|  | Diabetic microangiopathy | 1 ( 0.0%) | 0 ( 0.00%) | 1 ( 0.2%) | 0 ( 0.00%) | 0 ( 0.00%) |
|  | Embolism | 1 ( 0.0%) | 0 ( 0.00%) | 1 ( 0.2%) | 0 ( 0.00%) | 0 ( 0.00%) |
|  | Haematoma | 1 ( 0.0%) | 0 ( 0.00%) | 1 ( 0.2%) | 0 ( 0.00%) | 0 ( 0.00%) |
|  | Haemorrhage | 1 ( 0.0%) | 1 ( 0.2%) | 0 ( 0.00%) | 0 ( 0.00%) | 0 ( 0.00%) |
|  | Microangiopathy | 1 ( 0.0%) | 0 ( 0.00%) | 0 ( 0.00%) | 1 ( 0.2%) | 0 ( 0.00%) |
|  | Pelvic venous thrombosis | 1 ( 0.0%) | 0 ( 0.00%) | 1 ( 0.2%) | 0 ( 0.00%) | 0 ( 0.00%) |
|  | Poor peripheral circulation | 1 ( 0.0%) | 0 ( 0.00%) | 1 ( 0.2%) | 0 ( 0.00%) | 0 ( 0.00%) |
|  | Varicose vein | 1 ( 0.0%) | 0 ( 0.00%) | 1 ( 0.2%) | 0 ( 0.00%) | 0 ( 0.00%) |
|  | Venous thrombosis | 1 ( 0.0%) | 0 ( 0.00%) | 0 ( 0.00%) | 0 ( 0.00%) | 1 ( 0.5%) |
|  |  |  |  |  |  |  |
| Musculoskeletal and connective tissue disorders | All Patients | 37 ( 0.6%) | 14 ( 3.1%) | 18 ( 4.3%) | 3 ( 0.6%) | 3 ( 1.4%) |
|  | Osteoarthritis | 8 ( 0.1%) | 0 ( 0.00%) | 6 ( 1.4%) | 1 ( 0.2%) | 1 ( 0.5%) |
|  | Pain in extremity | 5 ( 0.1%) | 3 ( 0.7%) | 1 ( 0.2%) | 0 ( 0.00%) | 1 ( 0.5%) |
|  | Sjogren's syndrome | 5 ( 0.1%) | 3 ( 0.7%) | 1 ( 0.2%) | 1 ( 0.2%) | 0 ( 0.00%) |
|  | Arthralgia | 4 ( 0.1%) | 3 ( 0.7%) | 1 ( 0.2%) | 0 ( 0.00%) | 0 ( 0.00%) |
|  | Back pain | 3 ( 0.1%) | 1 ( 0.2%) | 2 ( 0.5%) | 0 ( 0.00%) | 0 ( 0.00%) |
|  | Bone pain | 2 ( 0.0%) | 2 ( 0.4%) | 0 ( 0.00%) | 0 ( 0.00%) | 0 ( 0.00%) |
|  | Musculoskeletal pain | 2 ( 0.0%) | 0 ( 0.00%) | 1 ( 0.2%) | 1 ( 0.2%) | 0 ( 0.00%) |
|  | Spinal column stenosis | 2 ( 0.0%) | 0 ( 0.00%) | 2 ( 0.5%) | 0 ( 0.00%) | 0 ( 0.00%) |
|  | Arthritis | 1 ( 0.0%) | 1 ( 0.2%) | 0 ( 0.00%) | 0 ( 0.00%) | 0 ( 0.00%) |
|  | Arthropathy | 1 ( 0.0%) | 0 ( 0.00%) | 1 ( 0.2%) | 0 ( 0.00%) | 0 ( 0.00%) |
|  | Bursitis | 1 ( 0.0%) | 0 ( 0.00%) | 1 ( 0.2%) | 0 ( 0.00%) | 0 ( 0.00%) |
|  | Intervertebral disc protrusion | 1 ( 0.0%) | 0 ( 0.00%) | 1 ( 0.2%) | 0 ( 0.00%) | 0 ( 0.00%) |
|  | Limb discomfort | 1 ( 0.0%) | 1 ( 0.2%) | 0 ( 0.00%) | 0 ( 0.00%) | 0 ( 0.00%) |
|  | Mobility decreased | 1 ( 0.0%) | 0 ( 0.00%) | 0 ( 0.00%) | 0 ( 0.00%) | 1 ( 0.5%) |
|  | Muscular weakness | 1 ( 0.0%) | 0 ( 0.00%) | 1 ( 0.2%) | 0 ( 0.00%) | 0 ( 0.00%) |
|  | Musculoskeletal discomfort | 1 ( 0.0%) | 1 ( 0.2%) | 0 ( 0.00%) | 0 ( 0.00%) | 0 ( 0.00%) |
|  | Musculoskeletal stiffness | 1 ( 0.0%) | 0 ( 0.00%) | 1 ( 0.2%) | 0 ( 0.00%) | 0 ( 0.00%) |
|  | Neck pain | 1 ( 0.0%) | 1 ( 0.2%) | 0 ( 0.00%) | 0 ( 0.00%) | 0 ( 0.00%) |
|  | Osteitis | 1 ( 0.0%) | 1 ( 0.2%) | 0 ( 0.00%) | 0 ( 0.00%) | 0 ( 0.00%) |
|  | Pain in jaw | 1 ( 0.0%) | 0 ( 0.00%) | 1 ( 0.2%) | 0 ( 0.00%) | 0 ( 0.00%) |
|  | Rheumatic disorder | 1 ( 0.0%) | 1 ( 0.2%) | 0 ( 0.00%) | 0 ( 0.00%) | 0 ( 0.00%) |
|  |  |  |  |  |  |  |
| Respiratory, thoracic and mediastinal disorders | All Patients | 33 ( 0.6%) | 6 ( 1.3%) | 21 ( 5.0%) | 4 ( 0.8%) | 7 ( 3.3%) |
|  | Dyspnoea | 10 ( 0.2%) | 0 ( 0.00%) | 5 ( 1.2%) | 1 ( 0.2%) | 4 ( 1.9%) |
|  | Cough | 7 ( 0.1%) | 4 ( 0.9%) | 1 ( 0.2%) | 2 ( 0.4%) | 0 ( 0.00%) |
|  | Pulmonary embolism | 7 ( 0.1%) | 0 ( 0.00%) | 5 ( 1.2%) | 0 ( 0.00%) | 2 ( 0.9%) |
|  | Chronic obstructive pulmonary disease | 5 ( 0.1%) | 0 ( 0.00%) | 4 ( 1.0%) | 1 ( 0.2%) | 0 ( 0.00%) |
|  | Pulmonary oedema | 5 ( 0.1%) | 0 ( 0.00%) | 4 ( 1.0%) | 1 ( 0.2%) | 1 ( 0.5%) |
|  | Pleural effusion | 2 ( 0.0%) | 0 ( 0.00%) | 2 ( 0.5%) | 0 ( 0.00%) | 0 ( 0.00%) |
|  | Respiratory failure | 2 ( 0.0%) | 0 ( 0.00%) | 1 ( 0.2%) | 0 ( 0.00%) | 1 ( 0.5%) |
|  | Bronchial disorder | 1 ( 0.0%) | 0 ( 0.00%) | 1 ( 0.2%) | 0 ( 0.00%) | 0 ( 0.00%) |
|  | Diaphragmatic disorder | 1 ( 0.0%) | 0 ( 0.00%) | 0 ( 0.00%) | 1 ( 0.2%) | 0 ( 0.00%) |
|  | Dysphonia | 1 ( 0.0%) | 1 ( 0.2%) | 0 ( 0.00%) | 0 ( 0.00%) | 0 ( 0.00%) |
|  | Dyspnoea at rest | 1 ( 0.0%) | 0 ( 0.00%) | 0 ( 0.00%) | 0 ( 0.00%) | 1 ( 0.5%) |
|  | Epistaxis | 1 ( 0.0%) | 0 ( 0.00%) | 1 ( 0.2%) | 0 ( 0.00%) | 0 ( 0.00%) |
|  | Hypercapnia | 1 ( 0.0%) | 0 ( 0.00%) | 0 ( 0.00%) | 1 ( 0.2%) | 0 ( 0.00%) |
|  | Hypoxia | 1 ( 0.0%) | 0 ( 0.00%) | 1 ( 0.2%) | 0 ( 0.00%) | 0 ( 0.00%) |
|  | Pleurisy | 1 ( 0.0%) | 1 ( 0.2%) | 0 ( 0.00%) | 0 ( 0.00%) | 0 ( 0.00%) |
|  | Pulmonary arterial hypertension | 1 ( 0.0%) | 0 ( 0.00%) | 0 ( 0.00%) | 0 ( 0.00%) | 1 ( 0.5%) |
|  |  |  |  |  |  |  |
| Gastrointestinal disorders | All Patients | 26 ( 0.4%) | 10 ( 2.2%) | 11 ( 2.6%) | 4 ( 0.8%) | 3 ( 1.4%) |
|  | Diarrhoea | 4 ( 0.1%) | 4 ( 0.9%) | 0 ( 0.00%) | 0 ( 0.00%) | 0 ( 0.00%) |
|  | Abdominal pain upper | 3 ( 0.1%) | 3 ( 0.7%) | 0 ( 0.00%) | 0 ( 0.00%) | 0 ( 0.00%) |
|  | Gastritis | 3 ( 0.1%) | 1 ( 0.2%) | 2 ( 0.5%) | 0 ( 0.00%) | 0 ( 0.00%) |
|  | Ascites | 2 ( 0.0%) | 0 ( 0.00%) | 2 ( 0.5%) | 0 ( 0.00%) | 0 ( 0.00%) |
|  | Gastric ulcer | 2 ( 0.0%) | 0 ( 0.00%) | 1 ( 0.2%) | 1 ( 0.2%) | 0 ( 0.00%) |
|  | Nausea | 2 ( 0.0%) | 2 ( 0.4%) | 0 ( 0.00%) | 1 ( 0.2%) | 0 ( 0.00%) |
|  | Pancreatitis | 2 ( 0.0%) | 0 ( 0.00%) | 2 ( 0.5%) | 0 ( 0.00%) | 0 ( 0.00%) |
|  | Vomiting | 2 ( 0.0%) | 0 ( 0.00%) | 1 ( 0.2%) | 1 ( 0.2%) | 0 ( 0.00%) |
|  | Dry mouth | 1 ( 0.0%) | 0 ( 0.00%) | 0 ( 0.00%) | 1 ( 0.2%) | 0 ( 0.00%) |
|  | Duodenal ulcer | 1 ( 0.0%) | 0 ( 0.00%) | 0 ( 0.00%) | 1 ( 0.2%) | 0 ( 0.00%) |
|  | Dysphagia | 1 ( 0.0%) | 0 ( 0.00%) | 1 ( 0.2%) | 0 ( 0.00%) | 0 ( 0.00%) |
|  | Gastrointestinal disorder | 1 ( 0.0%) | 1 ( 0.2%) | 0 ( 0.00%) | 0 ( 0.00%) | 0 ( 0.00%) |
|  | Gastrointestinal haemorrhage | 1 ( 0.0%) | 0 ( 0.00%) | 1 ( 0.2%) | 0 ( 0.00%) | 0 ( 0.00%) |
|  | Gastrointestinal inflammation | 1 ( 0.0%) | 0 ( 0.00%) | 0 ( 0.00%) | 1 ( 0.2%) | 0 ( 0.00%) |
|  | Gastrooesophageal reflux disease | 1 ( 0.0%) | 0 ( 0.00%) | 1 ( 0.2%) | 0 ( 0.00%) | 0 ( 0.00%) |
|  | Haematochezia | 1 ( 0.0%) | 0 ( 0.00%) | 0 ( 0.00%) | 0 ( 0.00%) | 1 ( 0.5%) |
|  | Ileus | 1 ( 0.0%) | 0 ( 0.00%) | 0 ( 0.00%) | 0 ( 0.00%) | 1 ( 0.5%) |
|  | Intestinal haemorrhage | 1 ( 0.0%) | 0 ( 0.00%) | 0 ( 0.00%) | 0 ( 0.00%) | 1 ( 0.5%) |
|  | Intestinal perforation | 1 ( 0.0%) | 0 ( 0.00%) | 1 ( 0.2%) | 0 ( 0.00%) | 0 ( 0.00%) |
|  | Large intestinal stenosis | 1 ( 0.0%) | 0 ( 0.00%) | 1 ( 0.2%) | 0 ( 0.00%) | 0 ( 0.00%) |
|  | Loose tooth | 1 ( 0.0%) | 1 ( 0.2%) | 0 ( 0.00%) | 0 ( 0.00%) | 0 ( 0.00%) |
|  | Volvulus | 1 ( 0.0%) | 0 ( 0.00%) | 0 ( 0.00%) | 0 ( 0.00%) | 1 ( 0.5%) |
|  |  |  |  |  |  |  |
| Metabolism and nutrition disorders | All Patients | 19 ( 0.3%) | 8 ( 1.8%) | 7 ( 1.7%) | 4 ( 0.8%) | 1 ( 0.5%) |
|  | Diabetes mellitus | 3 ( 0.1%) | 1 ( 0.2%) | 2 ( 0.5%) | 0 ( 0.00%) | 0 ( 0.00%) |
|  | Gout | 3 ( 0.1%) | 3 ( 0.7%) | 0 ( 0.00%) | 1 ( 0.2%) | 0 ( 0.00%) |
|  | Hypoglycaemia | 3 ( 0.1%) | 2 ( 0.4%) | 0 ( 0.00%) | 0 ( 0.00%) | 1 ( 0.5%) |
|  | Decreased appetite | 2 ( 0.0%) | 1 ( 0.2%) | 0 ( 0.00%) | 1 ( 0.2%) | 0 ( 0.00%) |
|  | Cachexia | 1 ( 0.0%) | 0 ( 0.00%) | 1 ( 0.2%) | 0 ( 0.00%) | 0 ( 0.00%) |
|  | Diabetic metabolic decompensation | 1 ( 0.0%) | 0 ( 0.00%) | 1 ( 0.2%) | 0 ( 0.00%) | 0 ( 0.00%) |
|  | Hypercholesterolaemia | 1 ( 0.0%) | 1 ( 0.2%) | 0 ( 0.00%) | 0 ( 0.00%) | 0 ( 0.00%) |
|  | Hyperglycaemia | 1 ( 0.0%) | 0 ( 0.00%) | 1 ( 0.2%) | 0 ( 0.00%) | 0 ( 0.00%) |
|  | Hyperlipidaemia | 1 ( 0.0%) | 0 ( 0.00%) | 0 ( 0.00%) | 1 ( 0.2%) | 0 ( 0.00%) |
|  | Hypernatraemia | 1 ( 0.0%) | 0 ( 0.00%) | 1 ( 0.2%) | 0 ( 0.00%) | 0 ( 0.00%) |
|  | Insulin resistance | 1 ( 0.0%) | 1 ( 0.2%) | 0 ( 0.00%) | 0 ( 0.00%) | 0 ( 0.00%) |
|  | Metabolic acidosis | 1 ( 0.0%) | 0 ( 0.00%) | 0 ( 0.00%) | 1 ( 0.2%) | 0 ( 0.00%) |
|  | Metabolic disorder | 1 ( 0.0%) | 0 ( 0.00%) | 1 ( 0.2%) | 0 ( 0.00%) | 0 ( 0.00%) |
|  | Underweight | 1 ( 0.0%) | 0 ( 0.00%) | 1 ( 0.2%) | 0 ( 0.00%) | 0 ( 0.00%) |
|  |  |  |  |  |  |  |
| Skin and subcutaneous tissue disorders | All Patients | 16 ( 0.3%) | 6 ( 1.3%) | 2 ( 0.5%) | 8 ( 1.6%) | 1 ( 0.5%) |
|  | Pruritus | 5 ( 0.1%) | 2 ( 0.4%) | 0 ( 0.00%) | 3 ( 0.6%) | 0 ( 0.00%) |
|  | Urticaria | 3 ( 0.1%) | 0 ( 0.00%) | 0 ( 0.00%) | 3 ( 0.6%) | 0 ( 0.00%) |
|  | Rash | 2 ( 0.0%) | 1 ( 0.2%) | 0 ( 0.00%) | 1 ( 0.2%) | 0 ( 0.00%) |
|  | Acne | 1 ( 0.0%) | 0 ( 0.00%) | 0 ( 0.00%) | 1 ( 0.2%) | 0 ( 0.00%) |
|  | Actinic keratosis | 1 ( 0.0%) | 1 ( 0.2%) | 0 ( 0.00%) | 0 ( 0.00%) | 0 ( 0.00%) |
|  | Cold sweat | 1 ( 0.0%) | 0 ( 0.00%) | 1 ( 0.2%) | 0 ( 0.00%) | 0 ( 0.00%) |
|  | Dermatitis allergic | 1 ( 0.0%) | 0 ( 0.00%) | 0 ( 0.00%) | 1 ( 0.2%) | 0 ( 0.00%) |
|  | Diabetic foot | 1 ( 0.0%) | 0 ( 0.00%) | 1 ( 0.2%) | 0 ( 0.00%) | 0 ( 0.00%) |
|  | Eczema nummular | 1 ( 0.0%) | 0 ( 0.00%) | 0 ( 0.00%) | 1 ( 0.2%) | 0 ( 0.00%) |
|  | Erythema | 1 ( 0.0%) | 0 ( 0.00%) | 0 ( 0.00%) | 0 ( 0.00%) | 1 ( 0.5%) |
|  | Fixed drug eruption | 1 ( 0.0%) | 0 ( 0.00%) | 0 ( 0.00%) | 1 ( 0.2%) | 0 ( 0.00%) |
|  | Hyperhidrosis | 1 ( 0.0%) | 0 ( 0.00%) | 0 ( 0.00%) | 1 ( 0.2%) | 0 ( 0.00%) |
|  | Nail bed inflammation | 1 ( 0.0%) | 1 ( 0.2%) | 0 ( 0.00%) | 0 ( 0.00%) | 0 ( 0.00%) |
|  | Papule | 1 ( 0.0%) | 0 ( 0.00%) | 0 ( 0.00%) | 1 ( 0.2%) | 0 ( 0.00%) |
|  | Pruritus generalised | 1 ( 0.0%) | 1 ( 0.2%) | 0 ( 0.00%) | 0 ( 0.00%) | 0 ( 0.00%) |
|  | Rash erythematous | 1 ( 0.0%) | 0 ( 0.00%) | 0 ( 0.00%) | 1 ( 0.2%) | 0 ( 0.00%) |
|  | Rosacea | 1 ( 0.0%) | 1 ( 0.2%) | 0 ( 0.00%) | 0 ( 0.00%) | 0 ( 0.00%) |
|  | Skin oedema | 1 ( 0.0%) | 0 ( 0.00%) | 0 ( 0.00%) | 1 ( 0.2%) | 0 ( 0.00%) |
|  | Skin swelling | 1 ( 0.0%) | 0 ( 0.00%) | 0 ( 0.00%) | 1 ( 0.2%) | 0 ( 0.00%) |
|  | Xanthelasma | 1 ( 0.0%) | 1 ( 0.2%) | 0 ( 0.00%) | 0 ( 0.00%) | 0 ( 0.00%) |
|  |  |  |  |  |  |  |
| Renal and urinary disorders | All Patients | 12 ( 0.2%) | 1 ( 0.2%) | 5 ( 1.2%) | 2 ( 0.4%) | 4 ( 1.9%) |
|  | Renal failure | 4 ( 0.1%) | 0 ( 0.00%) | 2 ( 0.5%) | 0 ( 0.00%) | 2 ( 0.9%) |
|  | Chronic kidney disease | 2 ( 0.0%) | 1 ( 0.2%) | 1 ( 0.2%) | 0 ( 0.00%) | 0 ( 0.00%) |
|  | Cystitis noninfective | 1 ( 0.0%) | 0 ( 0.00%) | 0 ( 0.00%) | 1 ( 0.2%) | 0 ( 0.00%) |
|  | Haematuria | 1 ( 0.0%) | 0 ( 0.00%) | 1 ( 0.2%) | 0 ( 0.00%) | 0 ( 0.00%) |
|  | Nephropathy | 1 ( 0.0%) | 0 ( 0.00%) | 1 ( 0.2%) | 0 ( 0.00%) | 0 ( 0.00%) |
|  | Renal artery stenosis | 1 ( 0.0%) | 0 ( 0.00%) | 0 ( 0.00%) | 0 ( 0.00%) | 1 ( 0.5%) |
|  | Renal cyst | 1 ( 0.0%) | 0 ( 0.00%) | 0 ( 0.00%) | 0 ( 0.00%) | 1 ( 0.5%) |
|  | Renal disorder | 1 ( 0.0%) | 0 ( 0.00%) | 0 ( 0.00%) | 1 ( 0.2%) | 0 ( 0.00%) |
|  | Renal embolism | 1 ( 0.0%) | 0 ( 0.00%) | 0 ( 0.00%) | 0 ( 0.00%) | 1 ( 0.5%) |
|  | Tubulointerstitial nephritis | 1 ( 0.0%) | 0 ( 0.00%) | 1 ( 0.2%) | 0 ( 0.00%) | 0 ( 0.00%) |
|  | Urethral stenosis | 1 ( 0.0%) | 0 ( 0.00%) | 1 ( 0.2%) | 0 ( 0.00%) | 0 ( 0.00%) |
|  |  |  |  |  |  |  |
| Psychiatric disorders | All Patients | 11 ( 0.2%) | 4 ( 0.9%) | 6 ( 1.4%) | 0 ( 0.00%) | 1 ( 0.5%) |
|  | Depression | 5 ( 0.1%) | 3 ( 0.7%) | 2 ( 0.5%) | 0 ( 0.00%) | 0 ( 0.00%) |
|  | Confusional state | 2 ( 0.0%) | 0 ( 0.00%) | 1 ( 0.2%) | 0 ( 0.00%) | 1 ( 0.5%) |
|  | Agitation | 1 ( 0.0%) | 1 ( 0.2%) | 0 ( 0.00%) | 0 ( 0.00%) | 0 ( 0.00%) |
|  | Alcohol abuse | 1 ( 0.0%) | 0 ( 0.00%) | 1 ( 0.2%) | 0 ( 0.00%) | 0 ( 0.00%) |
|  | Completed suicide | 1 ( 0.0%) | 0 ( 0.00%) | 1 ( 0.2%) | 0 ( 0.00%) | 0 ( 0.00%) |
|  | Disorientation | 1 ( 0.0%) | 0 ( 0.00%) | 0 ( 0.00%) | 0 ( 0.00%) | 1 ( 0.5%) |
|  | Restlessness | 1 ( 0.0%) | 0 ( 0.00%) | 1 ( 0.2%) | 0 ( 0.00%) | 0 ( 0.00%) |
|  |  |  |  |  |  |  |
| Hepatobiliary disorders | All Patients | 9 ( 0.2%) | 0 ( 0.00%) | 8 ( 1.9%) | 0 ( 0.00%) | 1 ( 0.5%) |
|  | Cholelithiasis | 4 ( 0.1%) | 0 ( 0.00%) | 4 ( 1.0%) | 0 ( 0.00%) | 0 ( 0.00%) |
|  | Cholecystitis | 2 ( 0.0%) | 0 ( 0.00%) | 2 ( 0.5%) | 0 ( 0.00%) | 0 ( 0.00%) |
|  | Liver disorder | 2 ( 0.0%) | 0 ( 0.00%) | 2 ( 0.5%) | 0 ( 0.00%) | 0 ( 0.00%) |
|  | Bile duct obstruction | 1 ( 0.0%) | 0 ( 0.00%) | 0 ( 0.00%) | 0 ( 0.00%) | 1 ( 0.5%) |
|  | Biliary colic | 1 ( 0.0%) | 0 ( 0.00%) | 1 ( 0.2%) | 0 ( 0.00%) | 0 ( 0.00%) |
|  |  |  |  |  |  |  |
| Ear and labyrinth disorders | All Patients | 8 ( 0.1%) | 5 ( 1.1%) | 2 ( 0.5%) | 1 ( 0.2%) | 0 ( 0.00%) |
|  | Vertigo | 6 ( 0.1%) | 4 ( 0.9%) | 1 ( 0.2%) | 1 ( 0.2%) | 0 ( 0.00%) |
|  | Hypoacusis | 1 ( 0.0%) | 1 ( 0.2%) | 0 ( 0.00%) | 0 ( 0.00%) | 0 ( 0.00%) |
|  | Tinnitus | 1 ( 0.0%) | 0 ( 0.00%) | 1 ( 0.2%) | 0 ( 0.00%) | 0 ( 0.00%) |
|  |  |  |  |  |  |  |
| Blood and lymphatic system disorders | All Patients | 7 ( 0.1%) | 1 ( 0.2%) | 5 ( 1.2%) | 0 ( 0.00%) | 1 ( 0.5%) |
|  | Anaemia | 3 ( 0.1%) | 0 ( 0.00%) | 3 ( 0.7%) | 0 ( 0.00%) | 0 ( 0.00%) |
|  | Iron deficiency anaemia | 2 ( 0.0%) | 1 ( 0.2%) | 1 ( 0.2%) | 0 ( 0.00%) | 0 ( 0.00%) |
|  | Polycythaemia | 1 ( 0.0%) | 0 ( 0.00%) | 0 ( 0.00%) | 0 ( 0.00%) | 1 ( 0.5%) |
|  | Thrombocytosis | 1 ( 0.0%) | 0 ( 0.00%) | 1 ( 0.2%) | 0 ( 0.00%) | 0 ( 0.00%) |
|  |  |  |  |  |  |  |
| Immune system disorders | All Patients | 5 ( 0.1%) | 3 ( 0.7%) | 1 ( 0.2%) | 1 ( 0.2%) | 0 ( 0.00%) |
|  | Allergy to chemicals | 1 ( 0.0%) | 0 ( 0.00%) | 0 ( 0.00%) | 1 ( 0.2%) | 0 ( 0.00%) |
|  | Hypersensitivity | 1 ( 0.0%) | 1 ( 0.2%) | 0 ( 0.00%) | 0 ( 0.00%) | 0 ( 0.00%) |
|  | Sarcoidosis | 1 ( 0.0%) | 0 ( 0.00%) | 1 ( 0.2%) | 0 ( 0.00%) | 0 ( 0.00%) |
|  | Seasonal allergy | 1 ( 0.0%) | 1 ( 0.2%) | 0 ( 0.00%) | 0 ( 0.00%) | 0 ( 0.00%) |
|  | Type IV hypersensitivity reaction | 1 ( 0.0%) | 1 ( 0.2%) | 0 ( 0.00%) | 0 ( 0.00%) | 0 ( 0.00%) |
| Social circumstances | All Patients | 5 ( 0.1%) | 2 ( 0.4%) | 2 ( 0.5%) | 1 ( 0.2%) | 0 ( 0.00%) |
|  | Refusal of treatment by patient | 2 ( 0.0%) | 2 ( 0.4%) | 0 ( 0.00%) | 0 ( 0.00%) | 0 ( 0.00%) |
|  | Death of relative | 1 ( 0.0%) | 0 ( 0.00%) | 0 ( 0.00%) | 1 ( 0.2%) | 0 ( 0.00%) |
|  | Device dependence | 1 ( 0.0%) | 0 ( 0.00%) | 1 ( 0.2%) | 0 ( 0.00%) | 0 ( 0.00%) |
|  | Walking disability | 1 ( 0.0%) | 0 ( 0.00%) | 1 ( 0.2%) | 0 ( 0.00%) | 0 ( 0.00%) |
|  |  |  |  |  |  |  |
| Not codable | Not codable | 3 ( 0.1%) | 0 ( 0.00%) | 0 ( 0.00%) | 2 ( 0.4%) | 1 ( 0.5%) |
|  | All Patients | 3 ( 0.1%) | 0 ( 0.00%) | 0 ( 0.00%) | 2 ( 0.4%) | 1 ( 0.5%) |
|  |  |  |  |  |  |  |
| Congenital, familial and genetic disorders | All Patients | 3 ( 0.1%) | 0 ( 0.00%) | 1 ( 0.2%) | 0 ( 0.00%) | 2 ( 0.9%) |
|  | Hypertrophic cardiomyopathy | 2 ( 0.0%) | 0 ( 0.00%) | 1 ( 0.2%) | 0 ( 0.00%) | 1 ( 0.5%) |
|  | Atrial septal defect | 1 ( 0.0%) | 0 ( 0.00%) | 0 ( 0.00%) | 0 ( 0.00%) | 1 ( 0.5%) |
|  |  |  |  |  |  |  |
| Endocrine disorders | All Patients | 3 ( 0.1%) | 2 ( 0.4%) | 0 ( 0.00%) | 1 ( 0.2%) | 0 ( 0.00%) |
|  | Hyperthyroidism | 3 ( 0.1%) | 2 ( 0.4%) | 0 ( 0.00%) | 1 ( 0.2%) | 0 ( 0.00%) |
|  |  |  |  |  |  |  |
| Product issues | All Patients | 3 ( 0.1%) | 1 ( 0.2%) | 2 ( 0.5%) | 0 ( 0.00%) | 0 ( 0.00%) |
|  | Device breakage | 1 ( 0.0%) | 1 ( 0.2%) | 0 ( 0.00%) | 0 ( 0.00%) | 0 ( 0.00%) |
|  | Device failure | 1 ( 0.0%) | 0 ( 0.00%) | 1 ( 0.2%) | 0 ( 0.00%) | 0 ( 0.00%) |
|  | Device material opacification | 1 ( 0.0%) | 0 ( 0.00%) | 1 ( 0.2%) | 0 ( 0.00%) | 0 ( 0.00%) |
|  |  |  |  |  |  |  |
| Reproductive system and breast disorders | All Patients | 2 ( 0.0%) | 0 ( 0.00%) | 2 ( 0.5%) | 0 ( 0.00%) | 0 ( 0.00%) |
|  | Cervical polyp | 1 ( 0.0%) | 0 ( 0.00%) | 1 ( 0.2%) | 0 ( 0.00%) | 0 ( 0.00%) |
|  | Prostatic disorder | 1 ( 0.0%) | 0 ( 0.00%) | 1 ( 0.2%) | 0 ( 0.00%) | 0 ( 0.00%) |
